# Development of the thermophilic fungus *Myceliophthora thermophila* into glucoamylase hyperproduction system via the metabolic engineering using improved AsCas12a variants

**DOI:** 10.1186/s12934-023-02149-4

**Published:** 2023-08-11

**Authors:** Zhijian Zhu, Manyu Zhang, Dandan Liu, Defei Liu, Tao Sun, Yujing Yang, Jiacheng Dong, Huanhuan Zhai, Wenliang Sun, Qian Liu, Chaoguang Tian

**Affiliations:** 1 Division of Life Sciences and Medicine, University of Science and Technology of China, Hefei, 230027 China; 2 Key Laboratory of Engineering Biology for Low-Carbon Manufacturing, Tianjin Institute of Industrial Biotechnology, Chinese Academy of Sciences, Tianjin, 300308 China; 3 National Technology Innovation Center of Synthetic Biology, Tianjin, 300308 China; 4 College of Biotechnology, Tianjin University of Science & Technology, Tianjin, 300457 China; 5 State Key Laboratory of Agrobiotechnology and MOA Key Laboratory of Soil Microbiology, College of Biological Sciences, China Agricultural University, Beijing, 100193 China

**Keywords:** *Myceliophthora thermophila*, Glucoamylase, Hyper-production, Genetic engineering, Secretion, CRISPR-AsCas12a, Morphology

## Abstract

**Background:**

Glucoamylase is an important enzyme for starch saccharification in the food and biofuel industries and mainly produced from mesophilic fungi such as *Aspergillus* and *Rhizopus* species. Enzymes produced from thermophilic fungi can save the fermentation energy and reduce costs as compared to the fermentation system using mesophiles. Thermophilic fungus *Myceliophthora thermophila* is industrially deployed fungus to produce enzymes and biobased chemicals from biomass during optimal growth at 45 °C. This study aimed to construct the *M. thermophila* platform for glucoamylase hyper-production by broadening genomic targeting range of the AsCas12a variants, identifying key candidate genes and strain engineering.

**Results:**

In this study, to increase the genome targeting range, we upgraded the CRISPR-Cas12a-mediated technique by engineering two AsCas12a variants carrying the mutations S542R/K607R and S542R/K548V/N552R. Using the engineered AsCas12a variants, we deleted identified key factors involved in the glucoamylase expression and secretion in *M. thermophila*, including *Mtstk-12*, *Mtap3m*, *Mtdsc-1* and *Mtsah-2*. Deletion of four targets led to more than 1.87- and 1.85-fold higher levels of secretion and glucoamylases activity compared to wild-type strain MtWT. Transcript level of the major amylolytic gene*s* showed significantly increased in deletion mutants. The glucoamylase hyper-production strain MtGM12 was generated from our previously strain MtYM6 via genetically engineering these targets *Mtstk-12*, *Mtap3m*, *Mtdsc-1* and *Mtsah-2* and overexpressing *Mtamy1* and *Mtpga3*. Total secreted protein and activities of amylolytic enzymes in the MtGM12 were about 35.6-fold and 51.9‒55.5-fold higher than in MtWT. Transcriptional profiling analyses revealed that the amylolytic gene expression levels were significantly up-regulated in the MtGM12 than in MtWT. More interestingly, the MtGM12 showed predominantly short and highly bulging hyphae with proliferation of rough ER and abundant mitochondria, secretion vesicles and vacuoles when culturing on starch.

**Conclusions:**

Our results showed that these AsCas12a variants worked well for gene deletions in *M. thermophila*. We successfully constructed the glucoamylase hyper-production strain of *M. thermophila* by the rational redesigning and engineering the transcriptional regulatory and secretion pathway. This targeted engineering strategy will be very helpful to improve industrial fungal strains and promote the morphology engineering for enhanced enzyme production.

**Supplementary Information:**

The online version contains supplementary material available at 10.1186/s12934-023-02149-4.

## Background

Polymers in ecosystems such as starch and lignocellulose first have to be degraded into small molecules before they can be taken up to apply as biofuel energy and simple sugars [1]. Filamentous fungi secrete different types and large amount of enzymes to degrade polysaccharides, for example glucoamylase, α-amylase, cellulase, xylanase and laccase [2]. Among these, the saprobic filamentous fungi, especially several *Aspergillus* and *Penicillium* species, *Trichoderma reesei* and *Myceliophthora thermophila* have crucial roles in fermentation industry biotechnology [3–6]. They have been wildly used as versatile microbial cell factories for producing valuable products, such as enzymes, organic acids, organic acids and antimicrobial compounds [7–9]. Classical UV and chemical mutagenesis have been used to enhance performance of hyper-producing strains, such as *T. reesei* RUT-C30 [10] and *A. niger* strain LDM3 [11].

Recent progress in genomic sequences and novel genetic manipulation methodologies, such as omics analysis and the CRISPR-Cas9/Cas12 system, have strongly expanded the synthetic biology toolkits of fungal strain engineering for enzymes hyper-production [12–14]. The efficient approaches for improvement of enzymes production are mainly manipulated by genetic engineering the gene transcription, mycelial morphology, metabolic network, and fungal secretory pathways [15]. For instance, overexpression of the key amylolytic gene regulator amyR in *M. thermophila* increased the amylase activity by 30% and deletion of amyR also resulted in 3-fold increase in CMCase and xylanase activities [16]. The CRISPR-HDR tool was used to knock in *goxC* gene expression cassettes into the precise loci of *glaA* and *amyA* to result in fourfold increased enzyme activity [17]. Combinatorial engineering of direct transcriptional activators ClrB, XlnR and AraR in *P. oxalicum* led to enhanced production of lignocellulolytic enzymes [18]. The deleting the Rho-GTPase racA gene of *A. niger* resulted in enhanced glucoamylase production, suggesting that the hyperbranching phenotype can be rationally exploited to obtain hypersecretion [19]. The overexpression of NADPH regeneration genes *gndA* and *maeA* in *A. niger* had a positive effect on glucoamylase production [20]. The knockout of the adaptor protein 3, which mediated the formation of vesicles and participated in intra-organelle membrane trafficking in eukaryotes, displayed cellulase hyper-production phenotypes in *Neurospora crassa* [21]. In *N. crassa* and *T. reesei*, the sterol regulatory element binding protein (SREBP) pathway negatively regulated protein secretion under lignocellulolytic conditions and the deletion of the key genes encoding components of the SREBP pathway, such as *tul-1* (*dsc-1*), *dsc-2* and *sah-2*, showed cellulase hyper-production phenotypes [22, 23].

*M. thermophila* is an industrially interested thermophilic fungus for biotechnological applications of high-temperature fermentations and production of thermostable carbohydrate-active enzymes (CAZymes) involved in biomass degradation [24–26]. Recently, the genomic editing tools including CRISPR-Cas9/Cas12a system and deaminase-cytosine base editors were developed by our group in *M. thermophila* [27–29] and have accelerated research progress on gene function, genetic engineering, and metabolic engineering [30, 31]. Soon after the establishment of CRISPR-Cas9/Cas12a system in *M. thermophila*, it was used for the improvement of the production of lignocellulolytic enzymes and generating the nonuple mutant M9 in which cellulase production was 9.0-fold higher than in the wild type [28]. More recently, the enhanced glucoamylase producing strain MtYM6 was generated through the flow cytometry-based plating-free system in *M. thermophila* [32]. However, the glucoamylase production level of the strain MtYM6 was much more lower as compared with the industrial glucoamylase-producing *A. niger* strains obtained by classical mutagenesis.

Since glucoamylase produced from thermophilic fungi can save the fermentation energy and reduce costs than the fermentation system from mesophiles, the new *M. thermophila* platform for glucoamylase hyper-production is in needed. In this study, we engineered two AsCas12a-RR and AsCas12a-RVR variants with altered PAM sequences to increase the genome targeting range. We applied this efficient editing tool of the AsCas12a variants to identify the four candidate factors involved in the glucoamylase expression and secretory pathway (*Mtstk-12*, *Mtap3m*, *Mtdsc-1* and *Mtsah-2*) and further monitored the phenotypes of the glucoamylase expression and secretion. We then significantly improved glucoamylase production in *M. thermophila* system based on our previous strain MtYM6 as host chassis through combinatorial engineering of these four factors and overexpressing a major α-amylase gene (Mycth_2305783) and G protein alpha subunit 3 (Mycth_2309848). Based on the efficient genetic manipulation, the glucoamylase hyper-producing strain MtGM12 was generated and investigated for transcriptional analysis and microscopic analyses for glucoamylase production.

## Results and discussion

### Improved AsCas12a variants increased the genomic targeting range in *M. thermophila*

Unlike CRISPR-Cas9 system, Cas12a uses only a single catalytic site to both cleave target double-stranded DNA and enables multiplex genes editing through the single customized CRISPR arrays [33]. However, one major constraint of Cas12a nuclease is their requirement for a longer PAM of the form 5′-TTTV-3′, where V can be A, C, or G. Recently, the Cas12a variants with altered PAM specificities were engineered and produced to successfully broaden the genome targeting range for gene editing [34, 35]. Previous work in 2017 by Gao et al. has engineered two AsCpf1 variants carrying the mutations S542R/K607R (referred to as RR) and S542R/K548V/N552R (referred to as RVR), which existed with altered PAM specificities including TYCV and TATV in human cells [34]. Recent study by Tóth et al. in 2000 has demonstrated that new PAM mutant variants of the four Cas12a nucleases that were active in mammalian and plant cells, including wild-type AsCas12a, LbCas12a, FnCas12a and MbCas12a orthologues and their mutant variants (RVR, RR, RVRR) [35]. The engineered AsCas12a-RVR and AsCas12a-RR variants were reported to exhibit TWTV (where W is A or T) and TYCV (where Y is C or T) PAM preferences as described in references 34 and 35, respectively. According to the knowledge previously discovered in these studies [34, 35], herein we have generated two AsCas12a variants carrying the mutations S542R/K607R (AsCas12a-RR) and S542R/K548V/N552R (AsCas12a-RVR) by using the AsCas12a expression plasmid previously constructed in *M. thermophila* [28] as a template. In this study, we tested for the first time whether the two AsCas12a mutations can efficiently loosen the long 5′-TTTV-3′ PAM constraints in this thermophilic fungus *M. thermophila*.

**Fig. 1:**
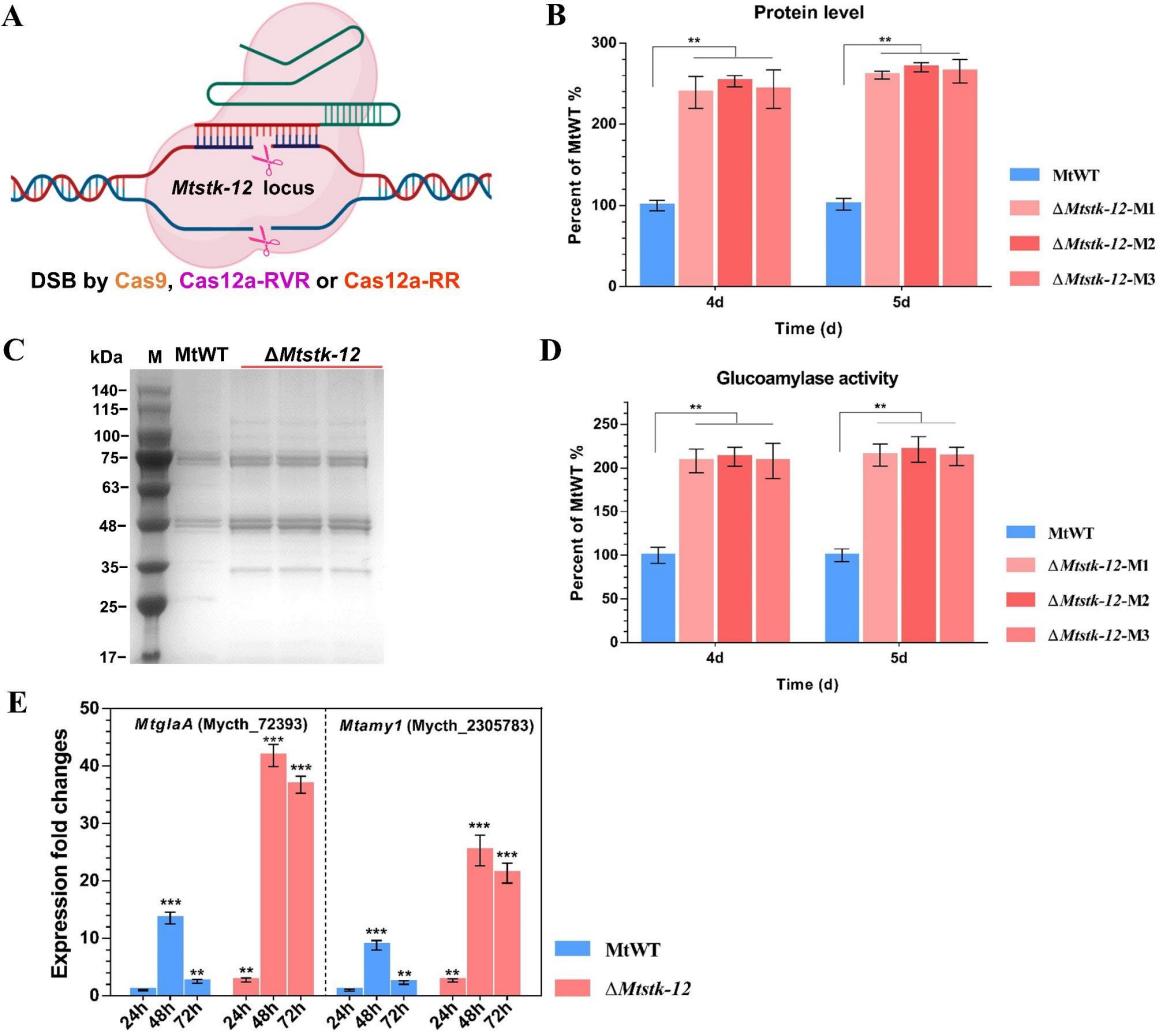
Effects of *Myceliophthora thermophila Mtstk-12* deletion on the amylolytic enzymes production. **(A)** Schematic view of the *Mtstk-12* disruption in *M. thermophila* using the CRISPR-Cas9 system. **(B)** Extracellular protein levels in culture supernatants of the ∆*Mtstk-12* mutants and wild-type MtWT strains after 4-day and 5-day incubating in starch. **(C)** SDS-PAGE analysis of the proteins secreted by *M. thermophila* strains after 5 days on starch medium. **(D)** Glucoamylase activity assay in the culture supernatants for the *M. thermophila* strains. **(E)** Transcript levels of the key glucoamylase gene *MtglaA* and α-amylase gene *Mtamy1* in the ∆*Mtstk-12* mutant relative to MtWT strain on starch. The data are normalized to the expression of the MtWT strain at 24 h for each tested gene, with actin gene (*Mycth_2314852*) expression levels used as an endogenous control in all samples. Error bars indicate the SD from three replicates

These AsCas12a-RR and AsCas12a-RVR-mediated editing system were applied throughout this work to facilitate loss-of-function of four target genes involved in the secretion pathway, including a protein kinase *Mtstk-12*, µ subunit of adaptor proteins *Mtap3m* and two sterol regulatory element binding protein (SREBP) pathway components *Mtdsc-1* and *Mtsah-2* (Fig. 1A and Additional files 4–7: Fig S1-S4). Here, we describe the efficiency results of the Cas system before detailing the rationale behind the targets and resulting secretion effects. The gene editing efficiencies and PAM preferences of two AsCas12a variants and Cas9 for all tested transformants are summarized in Table 1. The heterokaryotic phenotype in our PCR analysis (Additional files 4–7: Fig S1-S4) showed two bands of correct integration band and wild-type band. The heterozyogous clone remained stable heterokaryon after streaking to a single colony on new medium containing G418 or phosphinothricin. Due to the multinucleate nature of filamentous fungi, fungal protoplasts contained an uncertain number of nuclei, there are both homokaryotic and heterokaryotic clones often produced after transformation. Compared with the classic method, the efficiency of homologous recombination can be significantly improved in filamentous fungi by using the CRISPR/Cas9 system [12–14]. The deletion efficiencies of crRNA targeting of various PAM sequences were similarly high by using the AsCas12a-RR (68–95%) and AsCas12a-RVR (70–100%) variants as compared with the Cas9 (61–95%). Especially, the deletion frequency of the target *Mtdsc-1* was as high as 100% after co-transformation using the CRISPR-AsCas12a-RVR system and donor DNA, while the highly 95%knockout frequencies were also observed via the CRISPR-Cas9 and CRISPR-AsCas12a-RR systems. The disruption efficiency of the tested genes showed that the AsCas12a-RVR variant also had the high activity at TATV PAMs especially the TATG PAM (with 100% disruption efficiency of *Mtdsc-1*) and TATC PAM (with 90% disruption efficiency of *Mtsah-2*), and the AsCas12a-RR variant also efficiently cleaved TTCV and TCCV PAMs, such as TTCA PAM (with 95% disruption efficiency of *Mtdsc-1*) and TCCC PAM (with 85% disruption efficiency of *Mtsah-2*). Overall, these results demonstrate that the improved CRISPR-Cas12a system can efficiently mediate gene deletions with the expanded targeting range and be enable to target many previously inaccessible PAMs in *M. thermophila*.

**Table 1:** Summary of genome editing efficiency of the transformants by using the CRISPR/Cas9, CRISPR/Cas12a-RVR, and CRISPR/Cas12a-RR systems

Host strain^a^	Target genes	Elements in co-transformation	PAM sequences	No. of analysed Transformants	Gene disruption efficiency (%)
MtWT	*Mtstk-12*	Cas9 + sgRNA + donor	TGG	20	75
		Cas12a-RVR + crRNA + donor	TTTC	22	82
		Cas12a-RR + crRNA + donor	TTCG	22	77
MtWT	*Mtap3m*	Cas9 + sgRNA + donor	AGG	18	61
		Cas12a-RVR + crRNA + donor	TATA	20	70
		Cas12a-RR + crRNA + donor	TTCC	19	68
MtWT	*Mtdsc-1*	Cas9 + sgRNA + donor	CGG	20	95
		Cas12a-RVR + crRNA + donor	TATG	20	100
		Cas12a-RR + crRNA + donor	TTCA	20	95
MtWT	*Mtsah-2*	Cas9 + sgRNA + donor	CGG	20	75
		Cas12a-RVR + crRNA + donor	TATC	20	90
		Cas12a-RR + crRNA + donor	TCCC	20	85

^a^MtWT, *M. thermophila* wild type strain

### Deletion of *M. thermophila Mtstk-12* increased the starch-degrading enzymes production

Recently, a serine threonine protein kinase STK-12 was identified and functioned as a novel transcriptional brake which curbed cellulase gene expression in *N. crassa* [36]. The *N. crassa stk-12* disrupted mutant showed enhanced cellulase production under lignocellulolytic condition. Amylases are the starch-degrading enzymes that were the most important industrial enzymes with high biotechnological application [37]. To identify whether this brake role in amylases expression and production, we created the deletion mutant ∆*Mtstk-12* of its ortholog Mycth_2297068 in the important thermophilic fungus *M. thermophila* (Fig. 1A and Additional files 4: Fig S1). To assess the amylolytic phenotype of ∆*Mtstk-12*, we measured the extracellular total protein levels and enzymes activity of glucoamylase in culture supernatants of the *M. thermophila* wild-type (MtWT) strains and three independent homokaryotic ∆*Mtstk-12* mutants (∆*Mtstk-12*-M1 to ∆*Mtstk-12*-M3) after 4-day and 5-day fermentation on 2% starch medium. As shown in Fig. 1B, compared with the MtWT strain, the ∆*Mtstk-12* mutant secreted about 2.45- and 2.65-fold higher amounts of extracellular protein into the 2% starch medium after 4 and 5 days of cultivation. These data were further confirmed by the SDS-PAGE analysis (Fig. 1C). Consistent with this enhanced secreted protein production, the glucoamylase activity in the ∆*Mtstk-12* mutants were markedly increased about 2.10- and 2.16-fold higher than in the MtWT strain (Fig. 1D).

As described above, similar to the roles of *N. crassa* Stk-12 in the improvement of cellulase production, deletion of MtSTK-12 positively affected amylase proteins production. Since the STK-12 plays a critical role as a brake to turn down the transcription of cellulase genes in *N. crassa*, we hypothesized that MtSTK-12 may act as transcriptional regulator to control the expression of genes encoding amylolytic enzymes in *M. thermophila*. To test this hypothesis, we further monitored the expression levels of the major amylase genes, including the key glucoamylase gene (*Mycth_72393*, *MtglaA*) and α-amylase gene (*Mycth_2305783*, *Mtamy1*) [16, 31], in the MtWT and ∆*Mtstk-12* by real-time quantitative PCR (RT-qPCR). In both of the MtWT strain and ∆*Mtstk-12* mutant, two genes *MtglaA* and *Mtamy1* had their highest expression values after 48 h growth on starch (Fig. 1E). In the MtWT strain, the transcription levels of the *MtglaA* and *Mtamy1* were gradually increased and peaked at 48 h but rapidly declined at 72 h. This phenomenon is known as the feedback mechanism, termed repression under secretion stress (RESS), which selectively downregulates the transcription of genes encoding extracellular enzymes under endoplasmic reticulum (ER) stress to reduce ER load in filamentous fungi [38, 39]. In the ∆*Mtstk-12* mutant, however, the deletion of MtSTK-12 delayed the obvious repression of *MtglaA* and *Mtamy1* transcription. The transcript level of *MtglaA* and *Mtamy1* was significantly up-regulated 3.1-fold in the ∆*Mtstk-12* compared with the MtWT at 48 h and had not obviously decreased after 48 h and remained at a relatively high level at 72 h (Fig. 1E). The data indicated that MtSTK-12 played an important role as a brake to turn down the transcription of amylase genes in *M. thermophila*.

### Effect of the disruption of *Mtap3m* on amylolytic enzymes production in *M. thermophila*

**Fig. 2:**
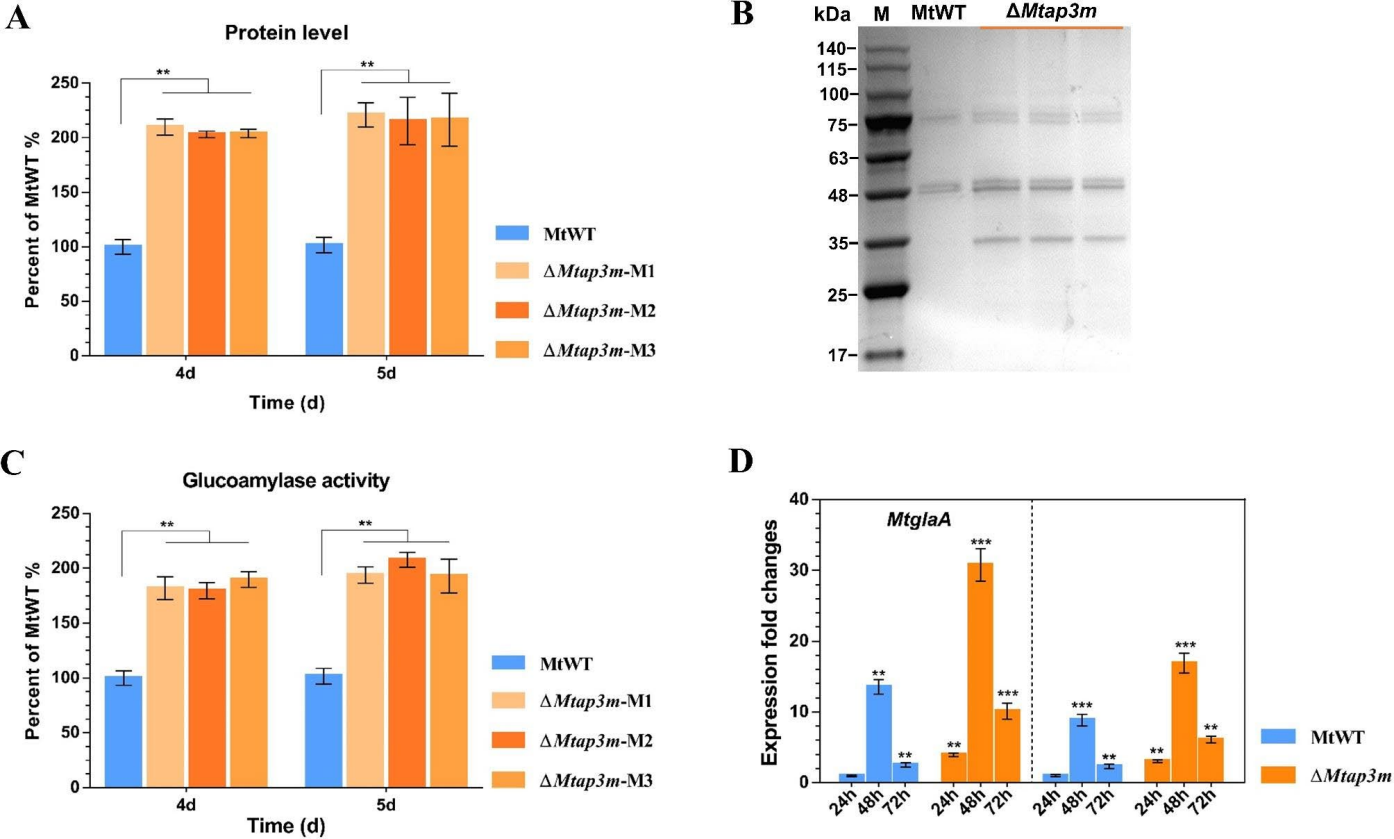
Deletion of *Mtap3m* increased the production of starch-degrading enzymes. **(A)** Assays for secreted protein concentration of the Δ*Mtap3m* mutants and MtWT strain in 2% starch medium after 4 and 5 days starch culture. **(B)** SDS-PAGE analysis of secreted protein of the Δ*Mtap3m* and MtWT strains after 5-days of incubation. **(C)** Glucoamylase activity assay in supernatants of 4- and 5-day starch-grown cultures of the Δ*Mtap3m* and MtWT strains. **(D)** Expression levels of the genes *MtglaA* and *Mtamy1* in the Δ*Mtap3m* compared to MtWT under starch condition by RT-qPCR analysis. Error bars indicate the SD from three replicates

Previous studies demonstrated that adaptor protein complexes (AP complexes) mediated the formation of vesicles and participate in intra-organelle membrane trafficking in eukaryotic cells [40, 41]. In *N. crassa*, the deletion of *Ncap3m* that encoded the µ subunit of the adaptor protein 3 (AP-3) complexes led to the significant increase in lignocellulase secretion under cellulosic conditions [21]. To study the function of the AP3m ortholog in the production of amylolytic enzymes in *M. thermophila*, the gene knockout mutant Δ*Mtap3m* was generated in *M. thermophila* using the CRISPR-Cas9 and AsCas12a variants editing system (Additional files 5: Fig S2). With starch as the sole carbon source, three independent *Mtap3m* deletion isolates showed increased levels of the secreted protein and glucoamylase activity compared with the parental strain MtWT. The extracellular protein concentrations in the Δ*Mtap3m* culture medium were 1.87- and 2.18- fold higher than those in parental strain MtWT cultures at 4 and 5 days of cultivation, respectively (Fig. 2A and B). The glucoamylase activities produced by the Δ*Mtap3m* mutants were approximately 185–198% higher as compared with the MtWT strain when both strains were cultured for 4‒5 days under starch conditions (Fig. 2C). These results indicated Mtap3m negatively regulated starch-degrading enzymes production in *M. thermophila*. This coincided with the previous observations of the *Ncap3m* deletion in *N. crassa*, in which has a significant impact on the secretion of lignocellulases [21]. To investigate the hypersecretion phenotype of the Δ*Mtap3m* mutants, we examined the expression levels of two major amylolytic genes in the MtWT and Δ*Mtap3m* strains using RT-qPCR analyses. Although the RESS phenomenon was observed in the Δ*Mtap3m* mutants, the transcription levels of the genes *MtglaA* and *Mtamy1* showed significantly up-regulated in the Δ*Mtap3m* mutant compared those in MtWT strain at all sampling time points of growth on starch (Fig. 2D). This higher transcription levels might be a reason for the higher secretion of amylolytic proteins in the Δ*Mtap3m* defect mutants.

### Identification of the SREBP pathway components *Mtdsc-1* and *Mtsah-2* that affected amylases production in *M. thermophila*

The sterol regulatory element binding protein (SREBP) pathway are conserved in eukaryotic cells and function in the regulation of sterol homeostasis [42, 43]. The Dsc-1 and Sah-2 are member elements of the Golgi apparatus E3 ligase complex and identified as the important components for SREBP pathway function [44, 45]. Recently, the cellulase hyper-production phenotype was observed in the both *N. crassa* and *T. reesei* mutant strains carrying a deletion of either *sah-2* or *dsc-1* [22, 23]. These studies in *N. crassa* and *T. reesei* demonstrated the SREBP pathway negatively regulates cellulases secretion under lignocellulolytic conditions. Since the homologs of SREBP pathway are conserved among filamentous fungi, it is possible that this hyper-production phenotype of components mutations in the SREBP pathway would be also identified in other fungi. To test this hypothesis, we constructed two knockout mutant strains ∆*Mtdsc-1* and ∆*Mtsah-2* of *dsc-1* and *sah-2* ortholog in *M. thermophila* for each (Additional file 6–7: Fig. S3-S4).

**Fig. 3:**
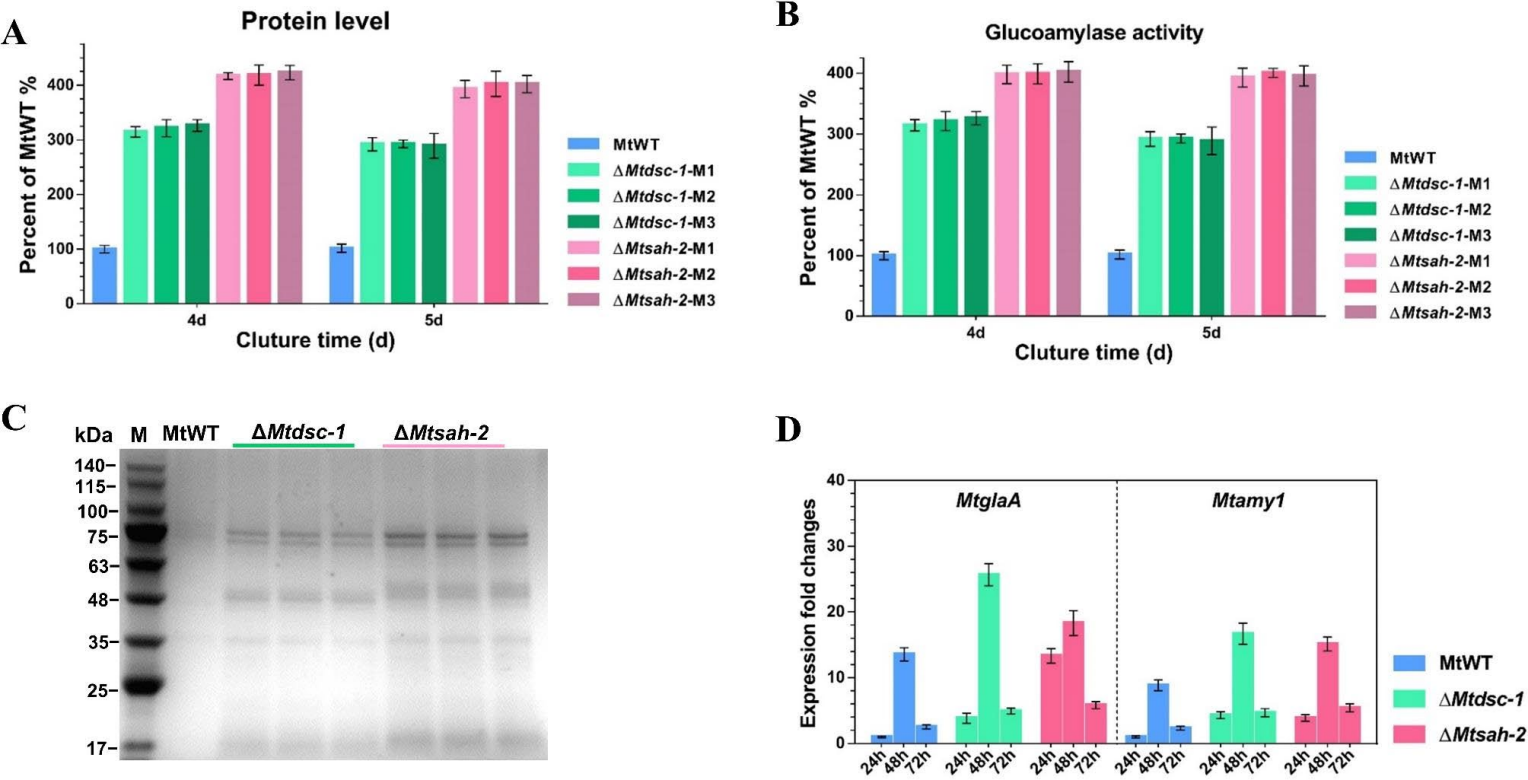
Hyper-production of amylolytic enzymes in *M. thermophila* due to deletion of two SREBP pathway components *Mtdsc‑1* and *Mtsah‑2*. **(A-B)** Assays for protein concentration and amylase activities of culture supernatants of the ∆*Mtdsc-1*, ∆*Mtsah-2* and MtWT strains after 4-days and 5-days shake-flask fermentation on starch. **(C)** SDS-PAGE analysis of secreted protein of the ∆*Mtdsc-1*, ∆*Mtsah-2* and MtWT strains after 5-days of incubation on starch. **(D)** Transcript levels of the genes *MtglaA* and *Mtamy1* in the ∆*Mtdsc-1*, ∆*Mtsah-2* and MtWT under starch condition by RT-qPCR analysis. **(E)** Transcript levels of the genes *MtglaA* and *Mtamy1* in the ∆*Mtdsc-1*, ∆*Mtsah-2* and MtWT after 24–72 h growth on starch medium. Error bars indicate the SD from three replicates

Each three independent homokaryotic ∆*Mtdsc-1* and ∆*Mtsah-2* deletions mutants were selected for amylase production analyses. As predicted, all three independently ∆*Mtdsc-1* and ∆*Mtsah-2* mutants displayed significantly higher secreted protein levels and enhanced glucoamylase activity after 4 d and 5d of incubation on starch as compared to the parental strain MtWT (Fig. 3A-C). The ∆*Mtdsc-1* mutants exhibited up to 2.91- and 2.77-fold increased amylases secretion and glucoamylase activity after 4 days of cultivation, while the ∆*Mtsah-2* mutants displayed a stronger pronounced increase in secreted protein levels (about 3.96-fold higher) and glucoamylase activity (about 3.21-fold) compared with the MtWT strain (Fig. 3A and B). Results of the SDS-PAGE analysis (Fig. 3C) of the extracellular proteins secreted by the MtWT, ∆*Mtdsc-1* and ∆*Mtsah-2* strains agreed with the protein levels and glucoamylase activities results. In the ∆*Mtdsc-1* and ∆*Mtsah-2* mutants, the relative expression levels of *MtglaA* and *Mtamy1* were significantly up-regulated compared to the MtWT stain at all sampling time points of growth on starch (Fig. 3D). Comparing with *Mtamy1*, the transcription level of *MtglaA* was quite high in the ∆*Mtsah-2* mutant in the first 24 h of incubation in the starch medium. These data coincided with the amylase hyper-production phenotype of the genes *Mtsah-2* and *Mtdsc-1* deletions.

### Combinational engineering of the brake MtStk-12, adaptor MtAp3m and main SREBP pathway components to generate *M. thermophila* glucoamylase hyper-production strains

Recent studies have demonstrated that the combinational genetic manipulation of transcriptional regulatory, secretory pathway, and light, photoreceptors and related signaling pathways of fungal strains was an efficient strategy to improve cellulolytic enzyme production in *T. reesei* [3, 15, 46], *P. oxalicum* [4, 34] and *M. thermophila* [27].

**Fig. 4:**
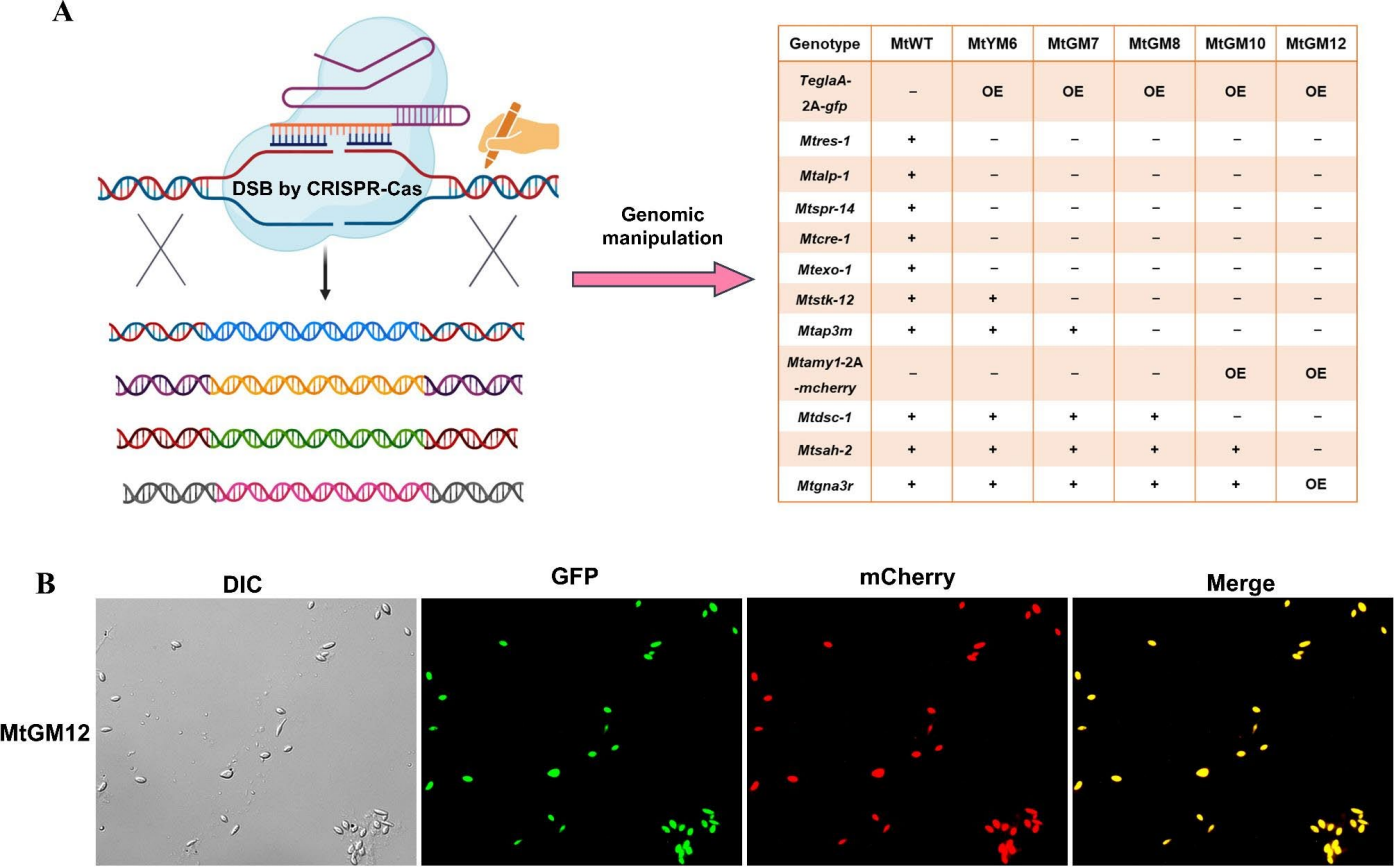
Construction of the glucoamylase hyperproducing strains via genetically engineering the gene expression, secretory pathway and the SREBP pathway by our CRISPR/Cas9-assisted marker recycling system. **(A)** Schematic illustration of the constructed strains MtGM7, MtGM8, MtGM10 and MtGM12 through iterative multiplex genome editing in consecutive steps in this study, including deleting four target genes *Mtstk-12*, *Mtap3m*, *Mtdsc‑1* and *Mtsah‑2* and overexpressing the genes *Mtamy1* and *Mtpga3*. **(B)** Microscopic fluorescence imaging of conidia of the obtained MtGM12 mutant. Scale bar represents 10 μm

Based on the results described above, in order to construct fungal strains hyper-producing glucoamylase, we started to genetically engineer our previously obtained strain MtYM6 [32] via our Camr technology (CRISPR/Cas-assisted marker recycling technology) [28]. After performance of successive three rounds of genomic manipulation, we generated the mutants MtGM7 (MtYM6 + Δ*Mtstk-12*), MtGM8 (MtYM6 + Δ*Mtstk-12*Δ*Mtap3m*), MtGM10 (MtYM6 + Δ*Mtstk-12*Δ*Mtap3m*Δ*Mtdsc-1*OE-*Mtamy1*) and MtGM12 (MtYM6 + Δ*Mtstk-12*Δ*Mtap3m*Δ*Mtdsc-1*OE-*Mtamy1*Δ*Mtsah-2*::*Mtpga3QL*) (Fig. 4A and Additional file 8–10: Fig. S5-S7). Bright visible both of green and red fluorescence was observed in the conidia of the MtGM12 mutant by microscopic analysis (Fig. 4B).

**Fig. 5:**
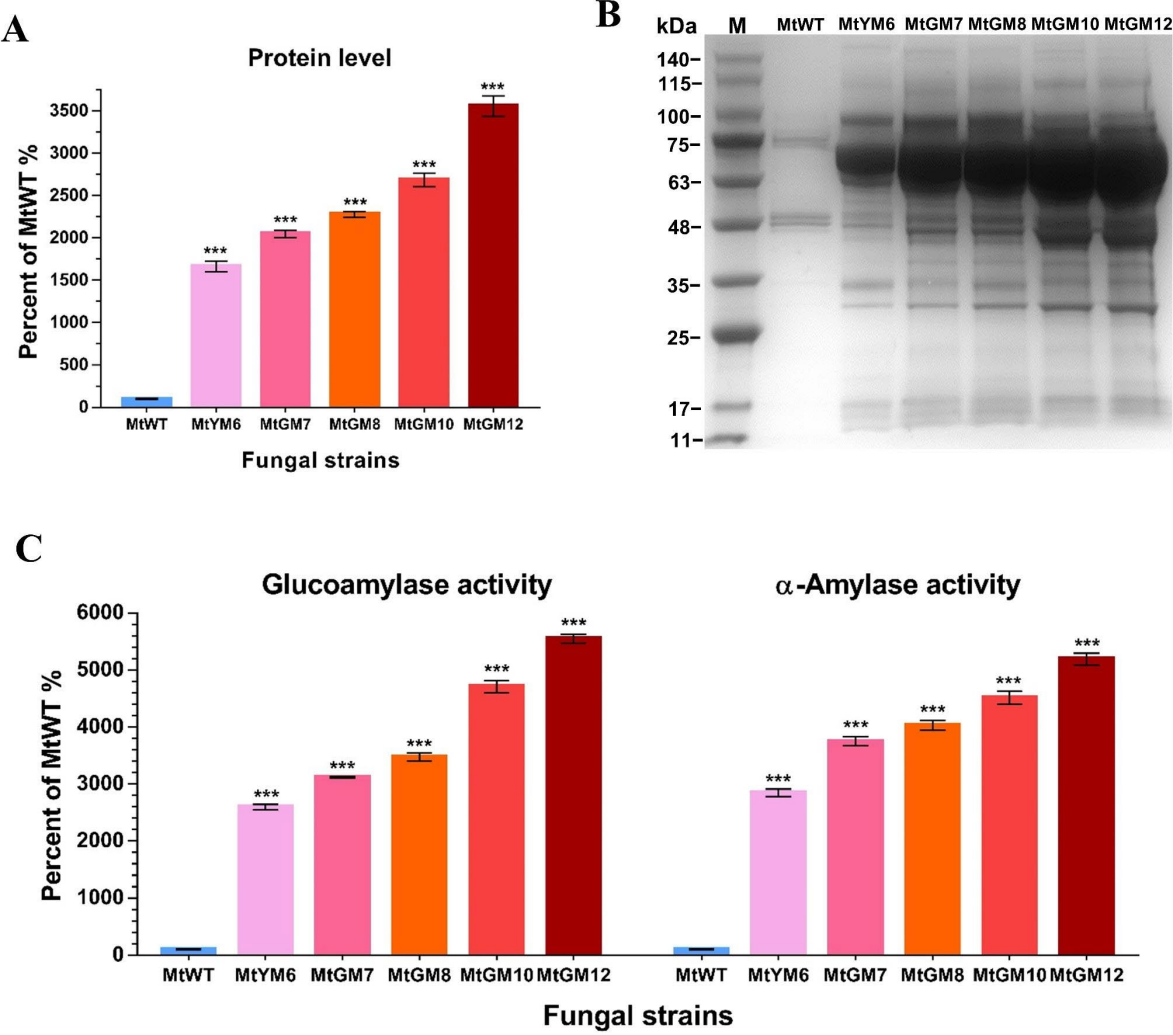
Amylolytic phenotypes analysis of the generated hyper-secretion mutants and MtWT strain. **(A)** Assays of total secreted protein in culture supernatants for all *M. thermophila* strains after 7-days culture in 2% starch medium supplemented with 0.75% yeast extract. **(B)** The sodium dodecylsulfate-polyacrylamide gel electrophoresis (SDS-PAGE) analysis of the proteins secreted by *M. thermophila* strains after 7 days on starch medium supplemented with 0.75% yeast extract. Extracellular proteins were collected from the culture supernatants secreted by the *M. thermophila* strains and proteins contained within an equivalent of 30 µL culture supernatants was resolved on a precast NuPAGE™ 4–12% gradient Bis-Tris gel, which was the commercially available NuPAGE electrophoresis system obtained from Thermo Fisher Scientific (Cat. No. NP0335BOX). Gels were stained using Coomassie blue. **(C)** The activities of glucoamylase and α-amylase in cultures supernatants of the *M. thermophila* strains after 7 days of cultivation in starch medium. Values are means ± SD (*n* = 3 repeats)

To assess the amylolytic hyperproduction phenotype, we compared the total secreted protein level, glucoamylases activity and α-amylases activity under starch conditions of the four generated mutants with those of the parental strain MtYM6 and the MtWT strain. As expected, these results showed that the secreted protein level and activities of glucoamylases and α-amylases of the MtGM7, MtGM8, MtGM10 and MtGM12 mutants were significantly higher than those of the parent strain MtYM6 (Fig. 5A to C). Strikingly, the MtGM12 mutant showed the most prominently hyper-production phenotype of amylolytic enzymes (~ 1255.8 mg L^− 1^), which was about 35.6-fold high than that of MtWT strain after 7 days of growth on 100 mL medium cultures with 2% starch as carbon source (Fig. 5A). Consistent with the significantly elevated level of the secreted protein and SDS-PAGE analysis, the associated amylolytic activities of the MtGM12 mutant were also significantly higher (by about 51.9‒55.5-fold) than those for the MtWT strain (Fig. 5C). These results demonstrated that simultaneous redesigning regulatory of the amylase expression, secretion and the SREBP pathway could altogether boost amylolytic enzymes production.

### Morphological analysis of MtWT strain and the engineered mutants

**Fig. 6:**
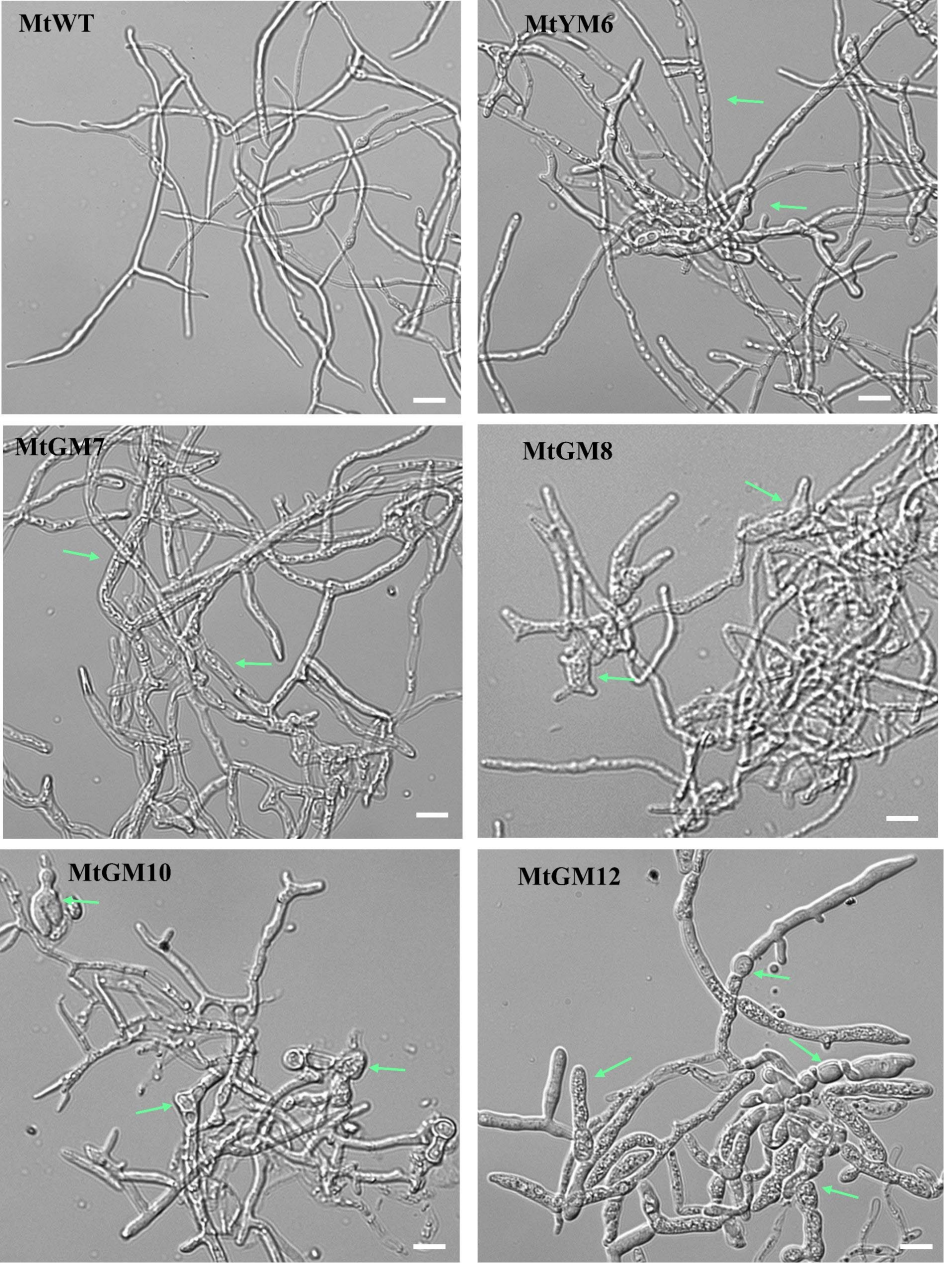
Fungal mycelial morphologies of the *M. thermophila* strains. The conidia from the amylolytic enzymes hyper-producing mutants MtGM7, MtGM8, MtGM10 and MtGM12, and the wild-type MtWT strain were separately inoculated into starch medium and batch-cultured for 5 days. Arrows indicate the short and highly bulging hyphae. Images were acquired under a OLYMPUS BX51 microscope using a QImaging Retiga 2000R camera. Scale bars = 10 μm

We further monitored whether the changes of mycelial morphologies were observed in the generated glucoamylase hyper-producing mutants as compared to the MtWT strain after 5-days of submerged fermentation in starch medium. The hyper-secretion mutants clearly showed marked morphological changes in the mycelial phenotypes relative to those in the MtWT strain (Fig. 6). Microscopic analyses revealed that the MtYM6 to MtGM8 mutants of produced some obvious bulging hyphae compared to the MtWT strain. Notably, the MtGM10 and MtGM12 mutants formed predominantly short and highly bulging hyphae (Fig. 6). All mutants exhibited significantly enhanced glucoamylase yields, especially the MtGM10 and MtGM12 which yields were 26.9- and 35.6-fold higher than that in the MtWT strain, respectively, indicating that highly swelling morphology favored amylolytic enzymes production. Future studies in *M. thermophila* will dissect the relationship between this morphological changes of bulging hyphae and hyper-production phenotype.

**Fig. 7:**
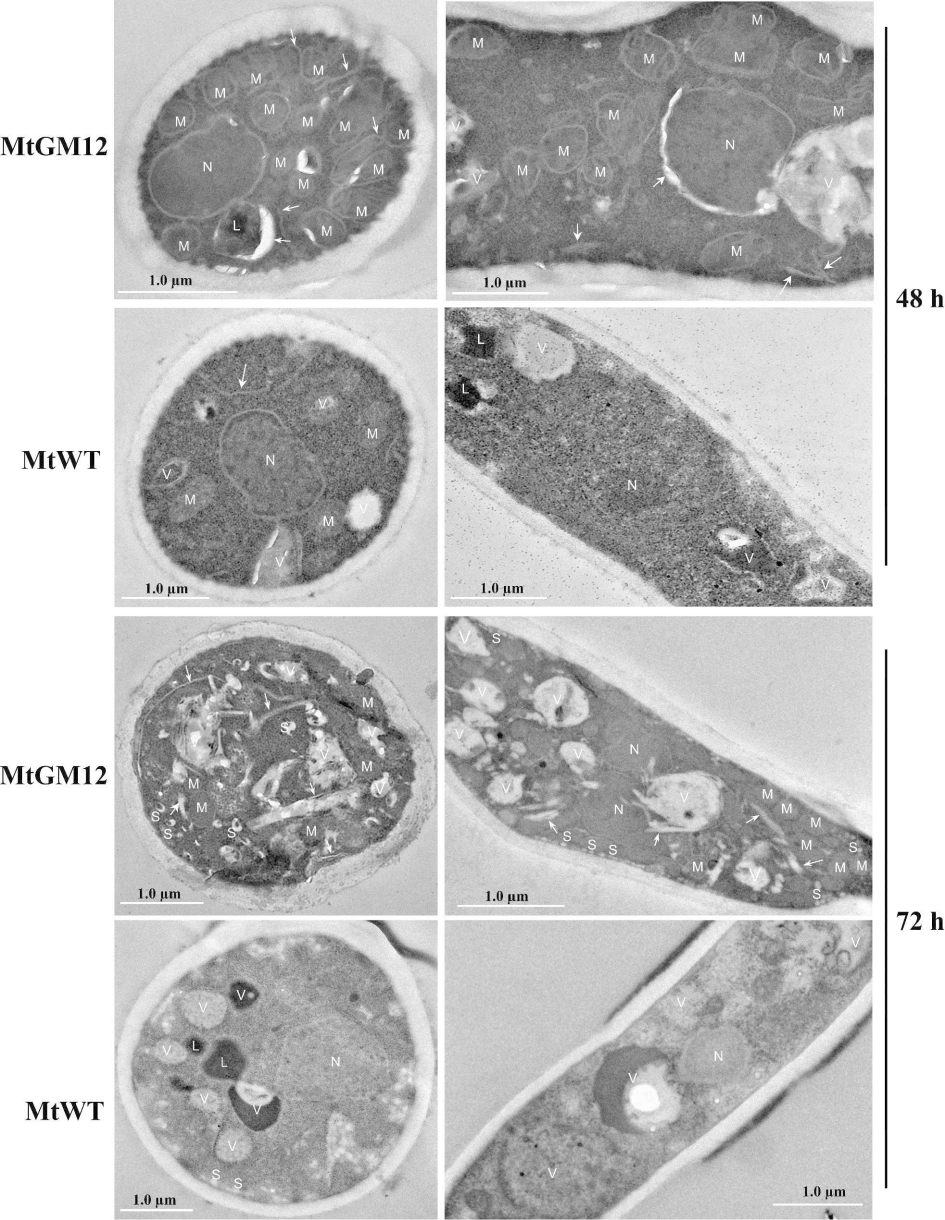
Transmission electron micrographs of the *M. thermophila* strains. The MtGM12 mutant and MtWT strain were grown in minimal medium with 2% (w/v) starch as sole carbon source for 48 or 72 h. Mycelia were collected and prepared for transmission electron microscopy. White arrows indicate endoplasmic reticulum. M, mitochondrion; N, nucleus; V, vacuole; S, secretion vesicles

Previous studies have demonstrated that the high protein secretion of the hypercellulolytic strain *T. reesei* RUT-C30 was found to be associated with changes in the ultrastructural characteristics as compared to the parent strain QM6a [47]. To further assess this possibility in the generated hyper-producing strain, through transmission electron microscopy (TEM), we analyzed the subcellular morphologies of the MtGM12 and MtWT strains when grown on starch for 4 days. In general, the MtGM12 strains looked morphologically different to MtWT cells during grown on starch medium for the same time period (Fig. 7 and Additional file 11: Fig. S8). As compared to the wild-type strain, the MtGM12 mutant displayed a dramatically proliferated endoplasmic reticulum (ER), in which plays important roles in protein synthesis and folding of the secretory process. When grown in starch medium for 48 h, the MtGM12 mutant hyphae contained large amounts of ER and increased numbers of mitochondria while comparably little ER was present in the MtWT hyphae at the same time (Fig. 7). Additionally, this phenomenon continued as fermentation progressed during 72-h (Figs. 7) and 96-h starch culture (Additional file 11: Fig. S8). The abundant mitochondria observed in the MtGM12 hyphae indicated that the MtGM12 cells might increase the number of mitochondria to supply more energy for gene expression and protein synthesis. In addition to ER and mitochondria, after 72 h and 96-h of growth in starch medium, mycelia from the MtGM12 mutant contained significantly increased amounts of secretion vesicles and vacuoles as compared to the MtWT. These changes might be one reason for the hyper-production of glucoamylase in MtGM12 mutant, implying that increased secretory activity is correlated with the proliferation of ER and increased numbers of mitochondria, secretion vesicles and vacuoles.

### Transcriptome analysis of the glucoamylase hyper-producing mutant MtGM12

**Fig. 8:**
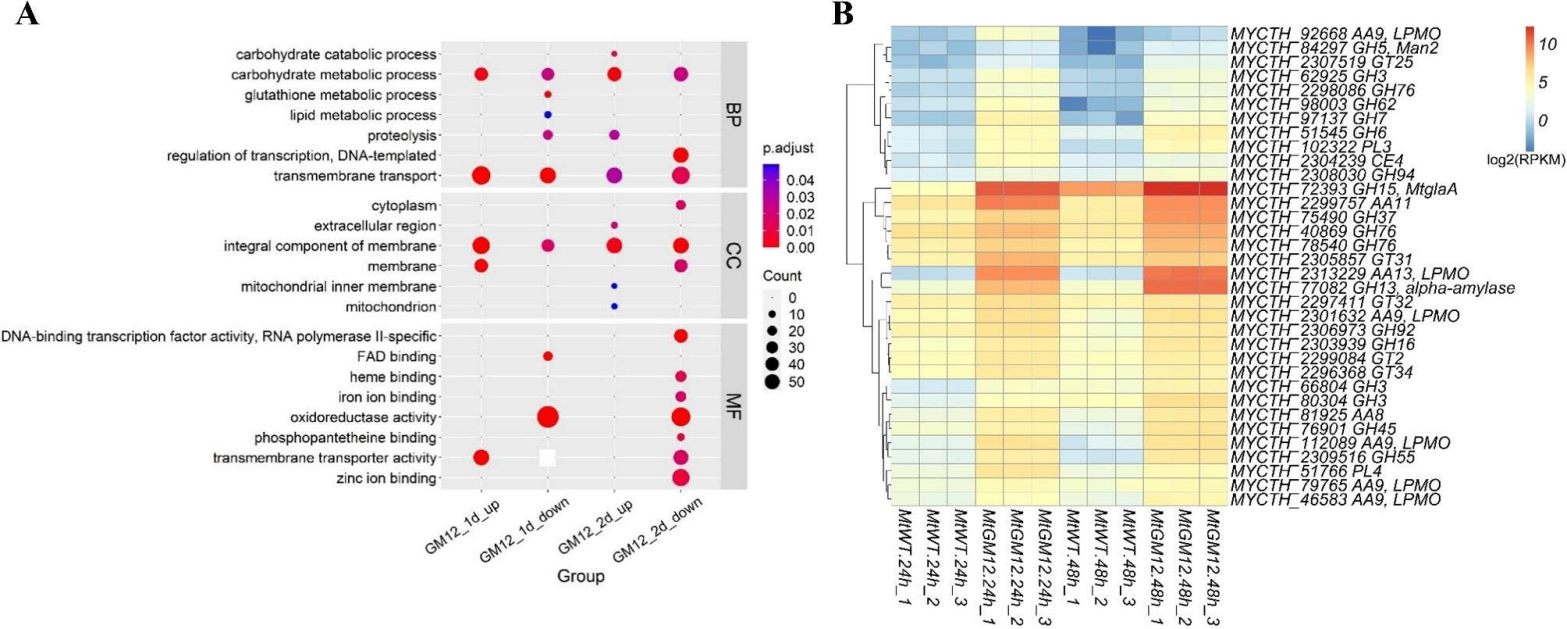
Comparative transcriptomics analysis of the MtGM12 mutant and MtWT strain grown in starch medium for 24 and 48 h. **(A)** Gene Ontology analysis of the up-regulated genes and down-regulated genes differentially expressed between the MtGM12 and MtWT strains on starch medium. **(B)** Heatmap analysis of expression profiles for the CAZy genes with statistically significant differences in transcript levels between the MtGM12 vs. MtWT under starch condition. Log-transformed expression values are color-coded

To investigate the role of the MtGM12 mutant on glucoamylases hyper-production, we compared the transcriptional profiles of the MtWT and MtGM12 strains during growth on starch medium by RNA-seq analysis (Additional file 2: Table S2). Expression pattern analyses revealed that 2426, and 2888 genes were differentially expressed between MtGM12 and MtWT after 24 and 48 h of incubation on starch conditions, respectively. The transcription levels of 1137 and 1269 of these genes were significantly up-regulated in the MtGM12 during growth on starch for 24 and 48 h. The mainly enriched Gene Ontology (GO) terms among the up-regulated genes were carbohydrate metabolic process, carbohydrate catabolic process, transmembrane transport, integral component of membrane, and transmembrane transporter activity (Fig. 8A and Additional file 3: Table S3). Expression pattern analyses revealed that 1289 and 1619 genes were repressed in the MtGM12 than MtWT when grown on starch for 24-h and 48-h, respectively. GO analysis showed that these down-regulated genes were over-represented in carbohydrate metabolic process, transmembrane transport, regulation of transcription DNA-templated, transmembrane transporter activity and oxidoreductase activity.

Analyses of the transcriptional profiles indicated that 34 carbohydrate active enzymes (CAZymes) genes involved in starch degradation were expressed at significantly higher levels in the MtGM12 than in MtWT after both 24-h and 48-h starch growth (Fig. 8B). These genes included the key glucoamylase gene (*Mycth_72393*, *MtglaA*), an alpha-amylase gene (*Mycth_77082*, GH13), a trehalase gene (*Mycth_75490*, GH37), two alpha-mannanase genes (*Mycth_40869* and *Mycth_78540*), three beta-glucosidase genes GH3 (*Mycth_62925*, *Mycth_80304* and *Mycth_66804*), and six LPMO genes (*Mycth_2313229*, *Mycth_2301632*, *Mycth_79765*, *Mycth_46583*, *Mycth_112089* and *Mycth_92668*). Additionally, 10 out of these 34 CAZymes, including MtGlaA, LPMOs and cellobiose dehydrogenase (Mycth_81925) and two pectate lyases (Mycth_102322, PL3 and Mycth_51766, PL4), showed overlapped with the ‘‘starch regulon’’, which we have recently identified in *M. thermophila* by systematic transcriptome analysis [16].

The glucoamylase, α-amylase and α-glucosidases are three main types of starch-degrading enzymes and have received much attention because of their industrial applications [48]. Our previous study of *M. thermophila* responding to soluble starch has demonstrated that the *Mycth_72393* was identified as the major glucoamylase gene and overexpression of *Mycth_72393* led to remarkable increase in amylase activity by 35% [16]. Recent studies have been reported that the starch-active polysaccharide monooxygenases (AA13, LPMOs) together with a biological redox partner, cellobiose dehydrogenase, oxidatively cleave α-glycosidic bonds of starch and could potentially be used with industrial amylases to convert starch into a fermentable carbohydrate [49, 50]. The expression levels of the major amylolytic genes such as the *Mycth_72393* and *Mycth_77082* and the starch-active LPMO *Mycth_2313229* were greatly increased about 10 − 89-fold, 23 − 121-fold and 488‒699-fold higher in the MtGM12 than in MtWT after 24-h and 48-h of growth on starch. The significantly elevated transcript abundances of these essential amylolytic genes in the MtGM12 were well consistent with the hyper-production results of amylolytic enzymes shown in Fig. 5. These data highlighting the *MtglaA*, alpha-amylase gene *Mycth_77082* and the starch-active LPMO *Mycth_2313229* as the key amylolytic genes target to engineer improving starch utilization in *M. thermophila*.

Previously, it was reported that the SREBP pathway in *N. crassa* regulated the expression of specific cellulase genes such as cellobiose dehydrogenase (AA3, CDH-1), lytic polysaccharide monooxygenases (AA9, LPMO) and genes involved in endoplasmic reticulum (ER) stress responses [23]. This ER stress triggers the unfolded protein response (UPR) to maintain protein homeostasis and cell survival or apoptosis. The UPR signaling pathway is highly conserved in fungi, in which it is mediated by protein endoribonuclease Ire1p (inositol-requiring enzyme 1) and the downstream basic leucine zipper (bZIP) transcription factor Hac1p [38, 39]. These results of *N. crassa* indicated a regulatory interplay of the SREBP and the UPR pathways might be also in other fungi. This findings led us to test the interconnections among the amylase secretion, the SREBP pathway and the UPR signaling pathway. We examined the expression level of six genes involved in UPR pathway were in the ∆*Mtdsc-1* and ∆*Mtsah-2* mutants, including *Mtgrp78* (Mycth_2315513), *Mtpdi1* (Mycth_2296005), *MtprpA* (Mycth_2295433), *Mtero-1* (Mycth_2313331), *Mtcnx1* (Mycth_2295005) and *Mtlhs1* (Mycth_2312968). The qRT-PCR results showed that the expression levels of these genes were higher in the ∆*Mtdsc-1* and ∆*Mtsah-2* mutants than in the MtWT strain (Additional file 7: Fig. S4D). We have further checked the tested six genes in the RNA-Seq data conducted on the final strain MtGM12. But we found that the expression level of only one tested gene *Mtlhs1* (Mycth_2312968, heat shock protein 70 family chaperone DnaK) was up-regulated in the final strain MtGM12 after 24 h of incubation on starch. That might be because of the previous deletion of the endoplasmic reticulum (ER) stress regulator MtRes-1 (Mycth_2302052) in the final strain MtGM12. However, some other genes involved the UPR pathway were significantly up-regulated in the MtGM12 compared with MtWT on starch condition in the RNA-Seq data, such as heat shock protein 40 DnaJ chaperone (Mycth_2088056 and Mycth_2304570), Golgi apparatus membrane protein TVP23 (Mycth_2296687) and TVP38 (Mycth_2295968), vacuolar protein sorting proteins Vps13 (Mycth_2305828), Vps27 (Mycth_2093728), Vps36 (Mycth_2310541) and Vps66 (Mycth_2090897), and Sect. 61 translocon beta subunit (Mycth_2073127), Sect. 63 Brl domain containing protein (Mycth_2299043). These data suggested that the cross talk of the SREBP and UPR pathways might be occurred in *M. thermophila*, as similar to what was displayed in *N. crassa*. Future work will be necessary to investigate the regulatory network mechanism of the UPR and SREBP pathways in filamentous fungi.

Taken together, these results demonstrated that rational redesigning and engineering the transcriptional regulatory, secretion pathway and the SREBP pathway enabled to construct the glucoamylase hyper-production platform in industrial thermophilic fungus.

## Conclusion

In this study, we upgraded the CRISPR-Cas12a-mediated technique by engineering two AsCas12a-RR and AsCas12a-RVR variants with altered PAM sequences to increase the genome targeting range. We applied this AsCas12a variants editing tool to identify the four candidate factors for improving glucoamylase production in important industrial thermophilic fungus *M. thermophila*. These factors included a transcriptional brake *Mtstk-12*, the µ subunit of adaptor protein complex *Mtap3m* and two SREBP pathway components *Mtdsc-1* and *Mtsah-2*. We further constructed the *M. thermophila* platform for glucoamylase hyper-production from our previously strain MtYM6 via genetically engineering four targets *Mtstk-12*, *Mtap3m*, *Mtdsc-1* and *Mtsah-2* and overexpressing *Mtamy1* and *Mtpga3*. The obtained hyperproducing MtGM12 mutant displayed the prominently hyper-production phenotypes of amylolytic enzymes with approximately 35.6-fold higher compared to MtWT after 7 days on starch. Interestingly, the MtGM12 formed predominantly short and highly bulging hyphae and displayed dramatically proliferated ER, abundant mitochondria, increased amounts of secretion vesicles and vacuoles. Consistent with these hyper-production results, RNA-seq data revealed that the major CAZymes genes involved in starch degradation were expressed at significantly higher levels in the MtGM12 than in MtWT. Together, the rational redesigning and engineering the transcriptional regulatory, secretion pathway and the SREBP pathway enable to construct the glucoamylase hyperproduction platform in *M. thermophila*. The application of the engineered strains will simplify the production process and increase production of glucoamylase, and add the value of product for starch biorefinery.

## Materials and methods

### Strains, media and growth conditions

*M. thermophila* ATCC 42,464 strain was obtained from the American Type Culture Collection (ATCC). The *M. thermophila* strains were grown on Vogel’s minimal medium supplemented with 2% sucrose (MM medium) at 45 °C for 10 days to obtain conidia. Antibiotics were added when needed for the screening of transformants. For vector manipulation and propagation, *Escherichia coli* DH5α was cultured at 37 °C in Luria–Bertani broth with kanamycin or ampicillin (100 µg mL^− 1^) for plasmid selection. In shaken-flask experiments for protein and enzyme assays and RNA extraction, *M. thermophila* conidia at 2.5 × 10^5^ spores per milliliter were inoculated in 100 mL of medium in a 250 mL Erlenmeyer flask (containing 1 × Vogel’s salt, 2% soluble starch, with or without 0.75% yeast extract) at 45 °C with shaking at 150 rpm.

### Construction of the plasmids for genetic engineering

All of the primer sequences used in this study are listed in Additional file 1: Table S1. All of the PCR products were amplified using Phusion high-fidelity DNA polymerase (Thermo Fisher, Waltham, MA, USA). To generate AsCas12a variants expression plasmids, two codon-optimized AsCas12a genes carrying the mutations S542R/K607R and S542R/K548V/N552R were synthesized by Life Sciences Research Services (Genewiz, Suzhou, China). with attached HAC-1 (GenPept: MYCTH_2310995) and SV40 nuclear localization signal (NLS-*Cas12a*-NLS) was synthesized artificially by the Life Science Research Services Company (Genewiz, Suzhou, China). For deletion, the crRNA expression cassettes and donor DNAs of the target genes were constructed as previously described [28]. The target genes were *Mtap3m* (Mycth_2307451), *Mtstk-12* (Mycth_2297068), *Mtdsc-1* (Mycth_2300902) and *Mtsah-2* (Mycth_2306768), as well as two markers *neo* (GenBank: HQ416708) and *bar* (GenBank: X17220). The 5′ and 3′ flanking sequences of target genes were amplified from *M. thermophila* genome. The selectable marker cassette P*trpC*-*neo* or P*trpC*-*bar* was amplified by PCR using the p0380-*neo* or p0380-*bar* plasmid [28] as the template. The *M. thermophila* U6 promoter-driven crRNA vector was created by overlapping PCR and cloned into the pJET1.2/blunt cloning vector for sequencing.

To construct the expression vector, the open reading frame of the α-amylase genes (Mycth_2305783, *Mtamy1*) and the sequence of His-2A-mCherry were amplified and cloned into pAN52-Ptef1-TtrpC using the Gibson Assembly® Cloning Kit (NEB, Beijing, China) to generate the plasmid Ptef1-amy-2A-mCherry-TtrpC. To overexpress the G protein alpha subunit 3 *Mtpga3* (Mycth_2309848) in the *Mtsah-2* locus, the 1500-bp promoter of *Mtpgk* (phosphoglycerate kinase, Mycth_2316240) and the coding sequence of the G protein α subunit Mtpga3QL (Q208L, dominantly activated PGA3 with substitution of Gln208 by leucine) were amplified separately. Two fragments and the 5’ and 3′ fragments of *Mtsah-2* were assembled and ligated into the pUC118 using Gibson kit to generate donor-*Mtsah-2*- MtPga3QL.

### Transformation of *M. thermophila* protoplasts

The PEG-mediated transformation of *M. thermophila* protoplasts was performed as described previously with some modifications [27]. For gene expression, 10 µg of linearized plasmid was transformed into fungal protoplasts as needed. For multiple gene editing by the CRISPR–Cas12a-RVR system, a mixture of 20 µg of PCR amplicons of the Ptef1-AsCas12aRVR-TtprC cassette, sgRNA expression cassettes, and the corresponding donor fragments of the target genes were mixed at the same molar concentration ratio and added to 200 µl of fungal protoplasts. The transformants were cultured on Vogel’s MM solid medium at 35 °C for 4 days with selection for resistance to geneticin (100 mg/L) or phosphinothricin (200 mg/L). The strains overexpressing specific protein domains or with disrupted genes were identified by diagnostic PCR.

### Protein, enzyme activity, and dry weight assay

Total extracellular protein in the supernatants after incubation was determined with a Bio-Rad DC protein assay kit (Bio-Rad, Hercules, CA, USA). Absorbance was measured at 595 nm and bovine serum albumin was used as the standard. For protein gel electrophoresis, a 30 µL of unconcentrated culture supernatant was loaded onto a polyacrylamide gel (Novex® NuPAGE® Precast 4–12% gradient Bis-Tris Protein Gels, Thermo Fisher Scientific, Cat. No. NP0335BOX) to examine the proteins by sodium dodecylsulfate polyacrylamide gel electrophoresis (SDS-PAGE). Gels were stained using Coomassie blue. Glucoamylase activity was determined by a modified 3, 5-dinitrosalicylic acid method using soluble starch as the substrate described in Li et al., 2020. The glucoamylase activity was evaluated by calculating the amount of reducing sugars released from starch hydrolysis from the absorbance at 540 nm. One unit of enzyme activity was defined as the amount associated with the release of 1 µmol glucose per minute under assay conditions. The α-amylase activity was determined by using the α-Amylase Assay Kit (Solarbio Life Sciences, Beijing, China, Cat#BC0615), in accordance with the manufacturer’s instructions. At the end of the time course experiments, fungal biomass dry weights of the cultures were estimated and then the fungi were harvested, dried, and weighed. The protein and enzyme activity values were further normalized by the mean of dry fungal biomass from biological replicates. All the estimates were performed in in triplicate assays.

### Microscopy and image processing

Mycelia samples were observed using the Olympus BX51 microscopy system and images were analyzed with Image-Pro Express 6.3 software. For transmission electron microscopy (TEM) analyses, the fungal samples were prepared by the by a previously described method [36]. Fungal mycelia were harvested and washed three times with PBS buffer. The collected mycelia were fixed in 0.1 M phosphate buffer containing 2.5% glutaraldehyde at 4˚C for 8 h, and then for 1 h in 1% osmium tetroxide at room temperature. The samples were gradually dehydrated in an ethanol series and embedded in LR White Resin. Ultrathin sections were stained with uranyl acetate for 30 min and then with lead citrate for 5 min. The stained sections were observed under the Hitachi HT7700 transmission electron microscope (Hitachi, Tokyo, Japan).

### Total RNAs extraction, quantitative real-time PCR and transcriptome analysis

The *M. thermophila* strains were inoculated into 1×Vogel’s salt with 2% starch and cultivated at 45 °C for 1–3 days of cultivation. The mycelia were harvested from 1-day, 2-day, and 3-day shake-flask cultures by vacuum filtration, and then homogenized in liquid nitrogen for total RNA extraction. The total RNA was extracted by using the TRIzol reagent (Invitrogen, Carlsbad, CA, USA) and purified using the Qiagen RNeasy Mini kit with digested DNase I (Qiagen, Hilden, Germany). Total RNA was synthesized to first strand cDNA using ReverTra Ace qPCR RT Kit (TOYOBO, Osaka, Japan). Quantitative real-time PCR (RT-qPCR) was performed using SYBR Green Real-time PCR Master Mix (TOYOBO, Osaka, Japan). Specific primers for RT-qPCR are listed in Supplementary Table 1. The actin gene (MYCTH_2314852) was used as an endogenous control for all experiments. The expression level of each gene was estimated using the 2^−ΔΔCT^ method [51].

RNA sequencing (RNA-seq), reads mapping, and quantification were accomplished according to the method described previously [9]. The purified RNA samples, with RNA Integrity Number > 8.0 determined using an Agilent 2100 Bioanalyzer (Agilent Technologies, Palo Alto, CA, USA), were sequenced by Novogene Corporation (Tianjin, China) to construct cDNA libraries and sequence using the Illumina Novaseq 6000 platform to generate 150-bp paired-end reads. All RNA-seq data in this study were generated by sequencing three independent biological replicates. Clean reads were mapped to the predicted transcripts for the *M. thermophila* ATCC 42,464 genome [24] with < 2-base mismatching, using TopHat (v2.0.12) [52]. Raw counts of reads uniquely mapped to only one gene were calculated for each gene by HTSeq-count (v0.13.5) [53] and used for normalizing transcript abundance (Reads Per Kilobase per Million mapped reads, RPKM) and analyzing differential gene expression using the DEseq2 package (v1.30.1) [54]. The criteria used to identify genes that were significantly differentially expressed between the wild-type and engineered strain were as follows: expression level fold-change > 2.0 (log_2_ ratio > 1 or < − 1) and DESeq adjusted *P* < 0.05.

### Statistical analysis

All of the experiments were carried out in three independent repeated assays. The statistical tests for significance were determined via one-way analysis of variance (ANOVA). For all tests, *P < 0.05, **P < 0.01, ***P < 0.001.

### Electronic supplementary material

Below is the link to the electronic supplementary material.

Additional file 1: Table S1. List of PCR primers used in this study

Additional file 2: Table S2. The transcriptional profiles of RNA-seq reads mapped to the genome of *M. thermophila* and the differential expression analysis

Additional file 3: Table S3. Gene Ontology (GO) enrichment analysis of the genes showing significantly differential expression in MtGM12 versus MtWT (*p*-values < 0.05)

Additional file 4: Figure S1 Schematic representation of genomic manipulation of the *Mtstk-12* knockout by the CRISPR–Cas9 (A), CRISPR/AsCas12a-RVR (B) and CRISPR/AsCas12a-RR (C) systems and identification of the gene deletion transformants by PCR analysis. The length of 0.9 kb represent negative, while the 1.8 kb represent the PCR products of positive *Mtstk-12* knockout strains, respectively

Additional file 5: Figure S2 Schematic representation of genomic editing of the *Mtap3m* deletion by the CRISPR–Cas9 (A), CRISPR/AsCas12a-RVR (B) and CRISPR/AsCas12a-RR (C) systems and identification of the gene deletion transformants by PCR analysis. The length of 1.6 kb represent negative, while the 2.4 kb represent the PCR products of positive *Mtap3m* knockout strains, respectively

Additional file 6: Figure S3 Schematic representation of gene deletion of the target *Mtdsc-1* by the CRISPR–Cas9 (A), CRISPR/AsCas12a-RVR (B) and CRISPR/AsCas12a-RR (C) systems and identification of the gene deletion transformants by PCR analysis. The length of 1.2 kb represent negative, while the 1.9 kb represent the PCR products of positive *Mtdsc-1* knockout strains, respectively

Additional file 7: Figure S4 Schematic representation of gene deletion of the target *Mtsah-2* by the CRISPR–Cas9 (A), CRISPR/AsCas12a-RVR (B) and CRISPR/AsCas12a-RR (C) systems and identification of the gene deletion transformants by PCR analysis. The length of 1.0 kb represent negative, while the 2.0 kb represent the PCR products of positive *Mtsah-2* disrupted strains, respectively. (D) RT-qPCR analyses of the essential genes involved in UPR pathway in the ∆*Mtdsc-1*, ∆*Mtsah-2*, and MtWT strains after 48 h of incubation on starch. Error bars indicate the SD from three replicates

Additional file 8: Figure S5 Schematic representation of the CRISPR/AsCas12aRVR-assisted marker recycling system for double genes knockout (A) and identification of the gene deletions of the *Mtstk-12* and marker *neo* in selected 20 transformants by PCR analysis (B). The expected lengths of knockout transformants of *Mtstk-12* and *neo* were 1.8 and 0.8 kb, respectively, while those of host strain (rightmost lane) was 0.9 and 2.0 kb, respectively. Heterokaryotic transformants showed two PCR bands (both of wild-type and knockout)

Additional file 9: Figure S6 Schematic view of genomic manipulation for genes knockout (A) and identification of *Mtap3m* and marker *bar* deletion transformants by PCR analysis (B). The 1.6 kb and 0.9 kb represent negative, 2.4 kb and 0.9 kb represent the PCR products of positive *Mtap3m* and *bar* knockout strains, respectively

Additional file 10: Figure S7 Schematic view of the CRISPR/AsCas12aRVR-based marker recycling system for genes knockout and knockin (A) and identification of the selected transformants by PCR analysis. The expected lengths of disrupted transformants of the *Mtdsc-1*, *Mtsah-2*, and *neo* were 1.9, 3.7, and 1.0 kb, respectively, while those of host strain (rightmost lane) was 1.2, 0.8 and 2.4 kb, respectively. The 2.6 kb represent the PCR products of positive overexpressing strains. Heterokaryotic transformants showed two PCR bands (both of wild-type and knockout)

Additional file 11: Figure S8 Transmission electron micrographs of the *M. thermophila* strains. The MtGM12 mutant and MtWT strain were grown in minimal medium with 2% (w/v) starch as sole carbon source for 24 or 96 h. Mycelia were collected and prepared for transmission electron microscopy. White arrows indicate endoplasmic reticulum. M, mitochondrion; N, nucleus; V, vacuole; S, secretion vesicles

## Data Availability

All data generated or analyzed during this study are included in this published article and its supplementary information files.

## Abbreviations

CRISPR: clustered regularly interspaced short palindromic repeats
Camr technology: CRISPR-Cas-assisted marker recycling technology
crRNA: CRISPR RNA
HDR: homology-directed repair
MtWT: *M. thermophila* wild type strain
CAZymes: carbohydrate-active enzymes
SREBP: sterol regulatory element binding protein
GlaA: glucoamylase
GO: Gene Ontology
LPMO: lytic polysaccharide monooxygenases
RESS: repression under secretion stress
ER: endoplasmic reticulum
TEM: transmission electron microscopy
RNA-seq: RNA sequencing
RPKM: Reads Per Kilobase per Million mapped reads

## Data availability

The RNA-seq raw data are available in the Gene Expression Omnibus database (accession number GSE227646) at the National Center for Biotechnology Information (NCBI).
