# Supplementary figures and images for: Development of the thermophilic fungus Myceliophthora thermophila into glucoamylase hyperproduction system via the metabolic engineering using improved AsCas12a variants
Source: Microb Cell Fact. 2023 Aug 11;22:150. doi: 10.1186/s12934-023-02149-4 (PMC10416393; doi:10.1186/s12934-023-02149-4)

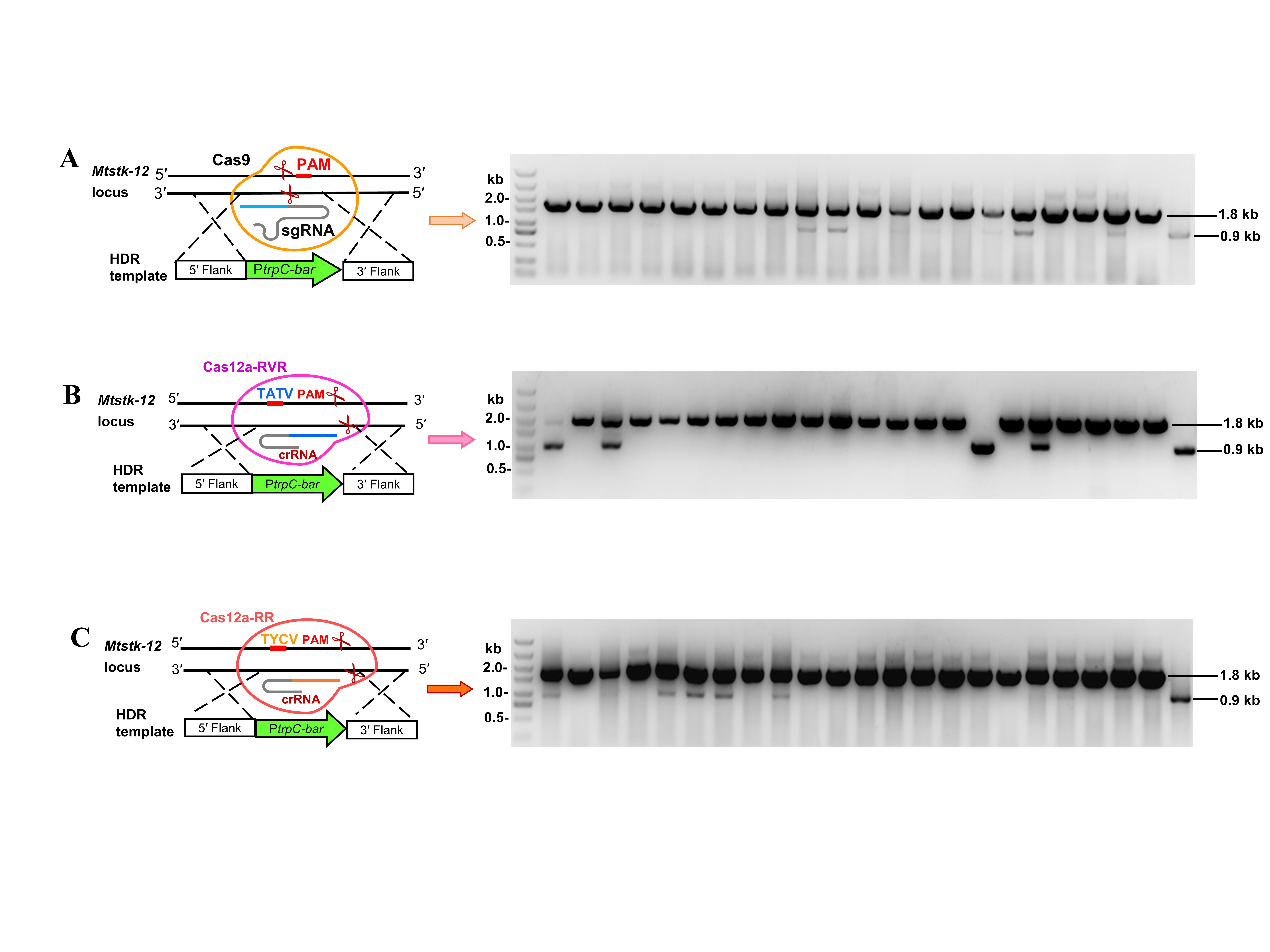

Supplement: Supplementary file 4 — Additional file 4: Figure S1 Schematic representation of genomic manipulation of the Mtstk-12 knockout by the CRISPR–Cas9 (A), CRISPR/AsCas12a-RVR (B) and CRISPR/AsCas12a-RR (C) systems and identification of the gene deletion transformants by PCR analysis. The length of 0.9 kb represent negative, while the 1.8 kb represent the PCR products of positive Mtstk-12 knockout strains, respectively [file 12934_2023_2149_MOESM4_ESM.jpg]

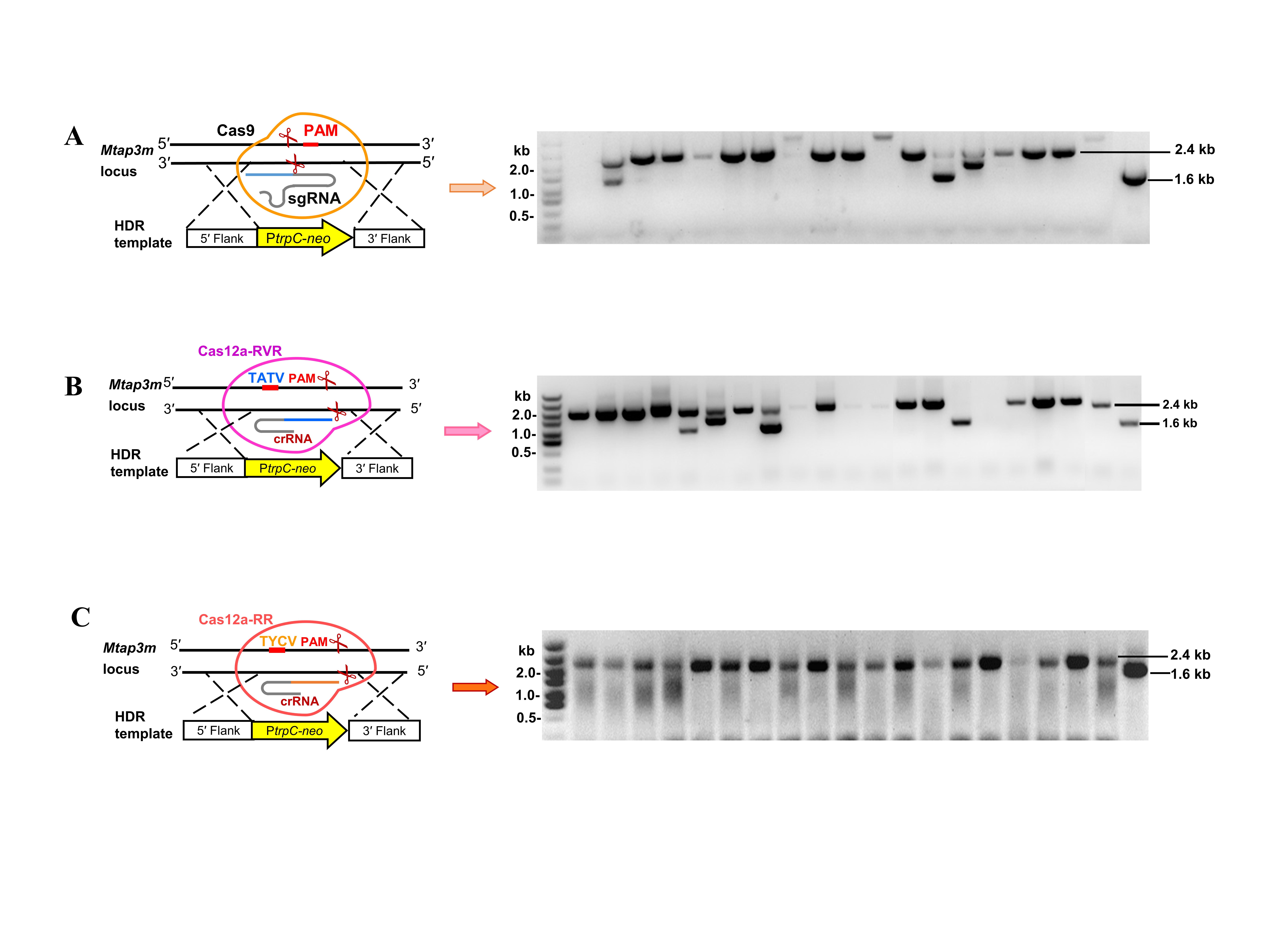

Supplement: Supplementary file 5 — Additional file 5: Figure S2 Schematic representation of genomic editing of the Mtap3m deletion by the CRISPR–Cas9 (A), CRISPR/AsCas12a-RVR (B) and CRISPR/AsCas12a-RR (C) systems and identification of the gene deletion transformants by PCR analysis. The length of 1.6 kb represent negative, while the 2.4 kb represent the PCR products of positive Mtap3m knockout strains, respectively [file 12934_2023_2149_MOESM5_ESM.jpg]

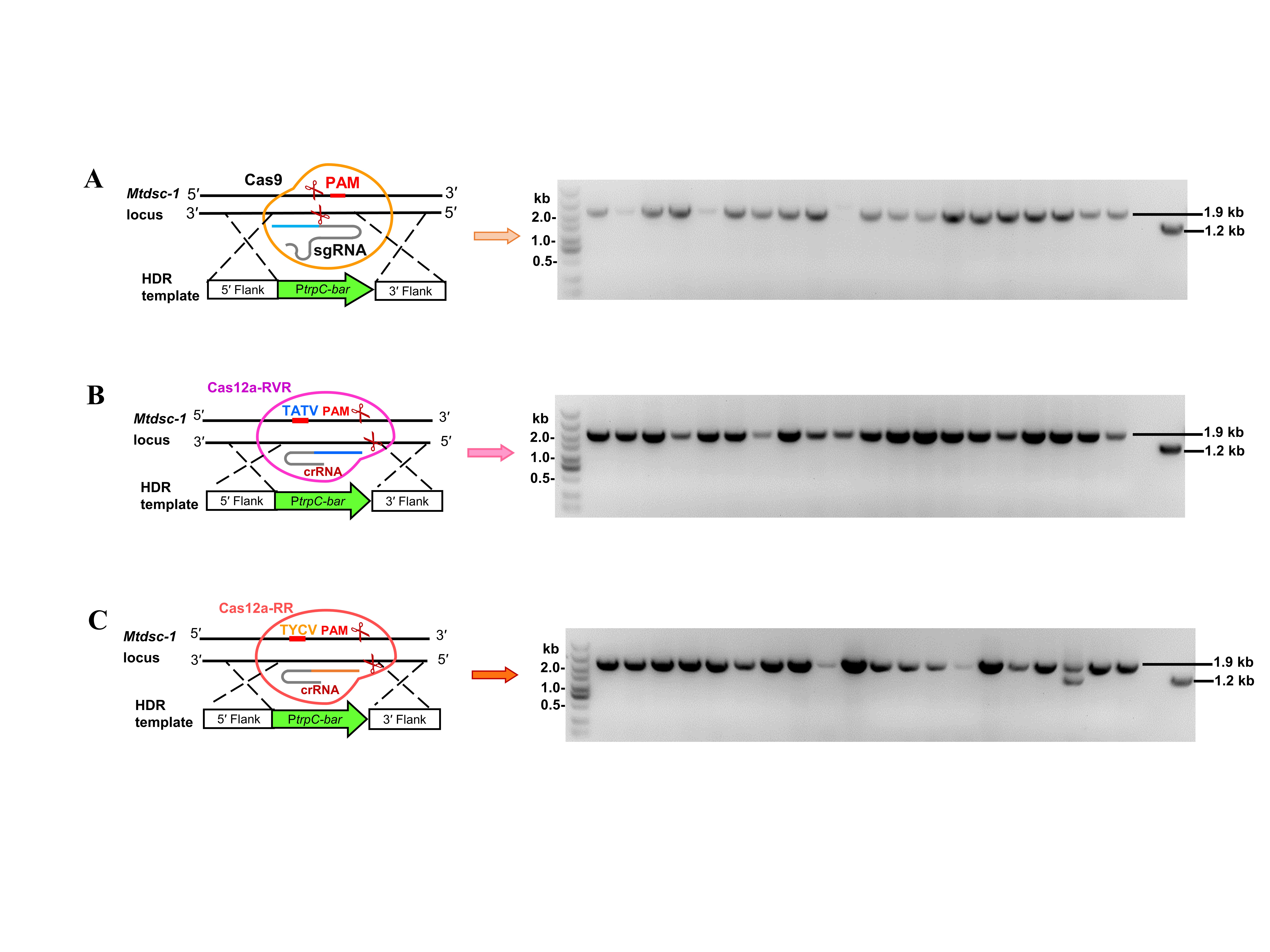

Supplement: Supplementary file 6 — Additional file 6: Figure S3 Schematic representation of gene deletion of the target Mtdsc-1 by the CRISPR–Cas9 (A), CRISPR/AsCas12a-RVR (B) and CRISPR/AsCas12a-RR (C) systems and identification of the gene deletion transformants by PCR analysis. The length of 1.2 kb represent negative, while the 1.9 kb represent the PCR products of positive Mtdsc-1 knockout strains, respectively [file 12934_2023_2149_MOESM6_ESM.jpg]

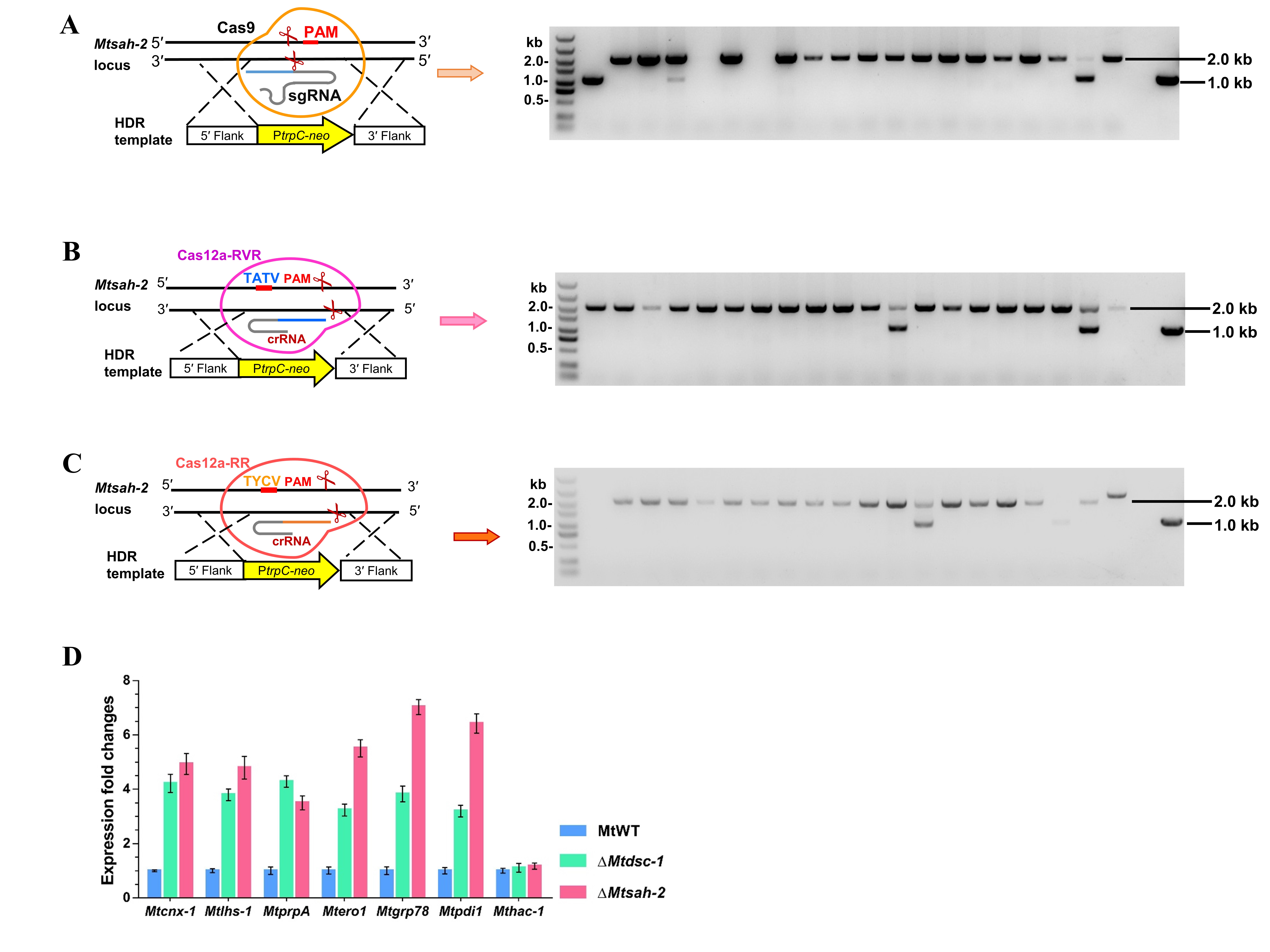

Supplement: Supplementary file 7 — Additional file 7: Figure S4 Schematic representation of gene deletion of the target Mtsah-2 by the CRISPR–Cas9 (A), CRISPR/AsCas12a-RVR (B) and CRISPR/AsCas12a-RR (C) systems and identification of the gene deletion transformants by PCR analysis. The length of 1.0 kb represent negative, while the 2.0 kb represent the PCR products of positive Mtsah-2 disrupted strains, respectively. (D) RT-qPCR analyses of the essential genes involved in UPR pathway in the ∆Mtdsc-1, ∆Mtsah-2, and MtWT strains after 48 h of incubation on starch. Error bars indicate the SD from three replicates [file 12934_2023_2149_MOESM7_ESM.jpg]

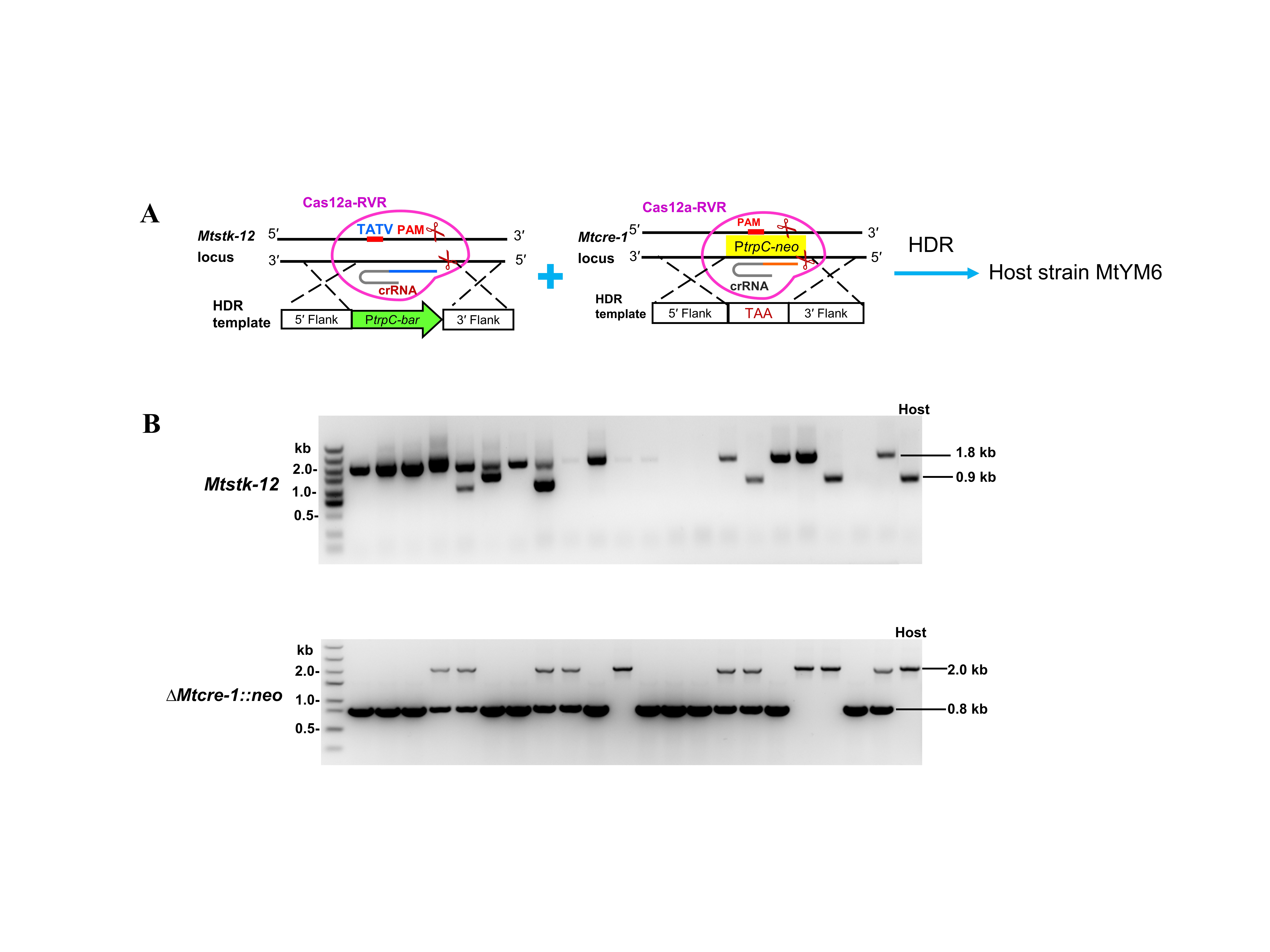

Supplement: Supplementary file 8 — Additional file 8: Figure S5 Schematic representation of the CRISPR/AsCas12aRVR-assisted marker recycling system for double genes knockout (A) and identification of the gene deletions of the Mtstk-12 and marker neo in selected 20 transformants by PCR analysis (B). The expected lengths of knockout transformants of Mtstk-12 and neo were 1.8 and 0.8 kb, respectively, while those of host strain (rightmost lane) was 0.9 and 2.0 kb, respectively. Heterokaryotic transformants showed two PCR bands (both of wild-type and knockout) [file 12934_2023_2149_MOESM8_ESM.jpg]

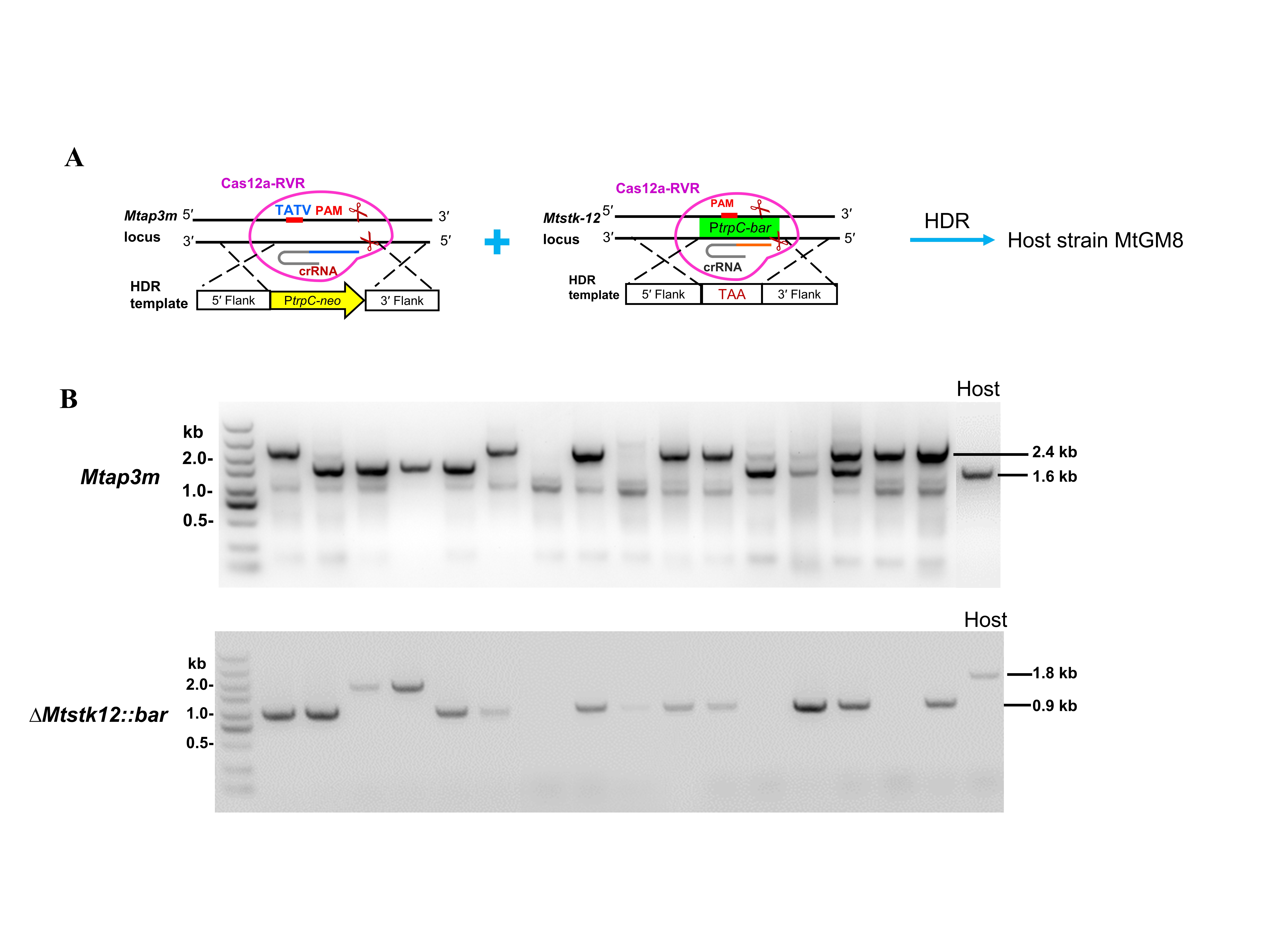

Supplement: Supplementary file 9 — Additional file 9: Figure S6 Schematic view of genomic manipulation for genes knockout (A) and identification of Mtap3m and marker bar deletion transformants by PCR analysis (B). The 1.6 kb and 0.9 kb represent negative, 2.4 kb and 0.9 kb represent the PCR products of positive Mtap3m and bar knockout strains, respectively [file 12934_2023_2149_MOESM9_ESM.jpg]

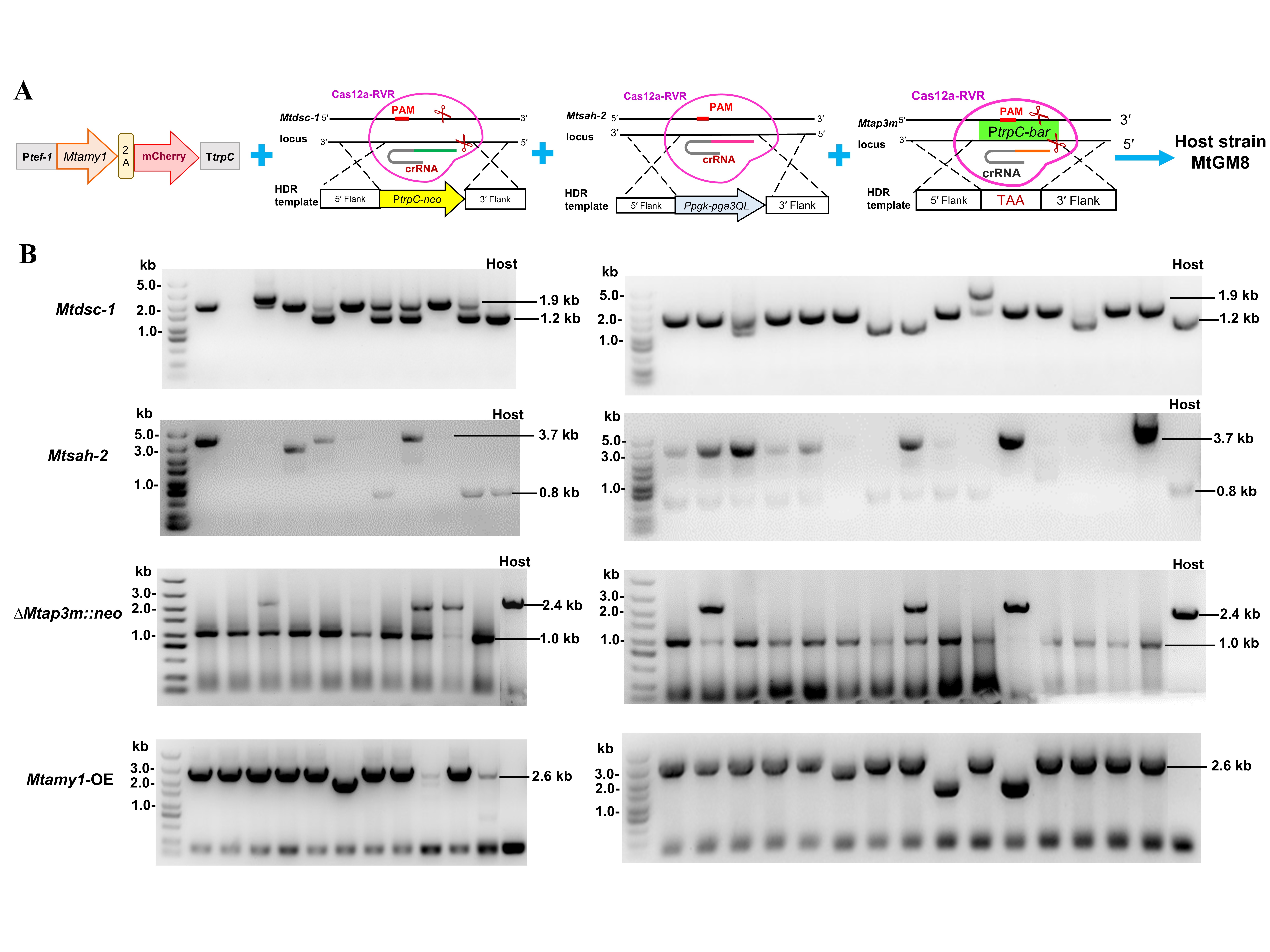

Supplement: Supplementary file 10 — Additional file 10: Figure S7 Schematic view of the CRISPR/AsCas12aRVR-based marker recycling system for genes knockout and knockin (A) and identification of the selected transformants by PCR analysis. The expected lengths of disrupted transformants of the Mtdsc-1, Mtsah-2, and neo were 1.9, 3.7, and 1.0 kb, respectively, while those of host strain (rightmost lane) was 1.2, 0.8 and 2.4 kb, respectively. The 2.6 kb represent the PCR products of positive overexpressing strains. Heterokaryotic transformants showed two PCR bands (both of wild-type and knockout) [file 12934_2023_2149_MOESM10_ESM.jpg]
